# Gravity-resisting colloidal collectives

**DOI:** 10.1126/sciadv.ade3161

**Published:** 2022-11-18

**Authors:** Junhui Law, Hui Chen, Yibin Wang, Jiangfan Yu, Yu Sun

**Affiliations:** ^1^ School of Science and Engineering, The Chinese University of Hong Kong, Shenzhen, China.; ^2^ Shenzhen Institute of Artificial Intelligence and Robotics for Society, Shenzhen, China.; ^3^ Department of Mechanical and Industrial Engineering, University of Toronto, Toronto, Canada.; ^4^ Institute of Biomedical Engineering, University of Toronto, Toronto, Canada.; ^5^ Department of Electrical and Computer Engineering, University of Toronto, Toronto, Canada.; ^6^ Department of Computer Science, University of Toronto, Toronto, Canada.; ^7^ Robotics Institute, University of Toronto, Toronto, Canada.

## Abstract

Self-assembly of dynamic colloidal structures along the vertical direction has been challenging because of gravity and the complexity in controlling agent-agent interactions. Here, we present a strategy that enables the self-growing of gravity-resisting colloidal collectives. By designing a unique dual-axis oscillating magnetic field, time-varying interparticle interactions are induced to assemble magnetic particles against gravity into vertical collectives, with the structures continuing to grow until reaching dynamic equilibrium. The collectives have swarm behavior and are capable of height reconfiguration and adaptive locomotion, such as moving along a tilted substrate and under nonzero fluidic flow condition, gap and obstacle crossing, and stair climbing.

## Teaser

Magnetic particles self-assemble into dynamic, gravity-resisting vertical collectives in an oscillating magnetic field.

## INTRODUCTION

Self-assembly is a process in which individual agents autonomously arrange themselves into collective structures (*1*). While the formation of static structures is through energy minimization, dynamic collectives actively consume energy to gain structural complexity and function diversity (*1*–*4*). Dynamic colloidal self-assembly is an essential means of creating functional materials and systems to enable applications in materials engineering such as the formation of intelligent matters (*5*), in chemical engineering such as catalysis (*6*, *7*), in microfactories such as contactless material handling (*8*–*10*) and the construction of photonic crystals (*11*, *12*), and in health care such as targeted delivery (*13*–*16*) and therapeutics (*17*).

Recent advances in self-assembly of dynamic colloidal structures are achieved through constructing programmable interparticle bonds, such as DNA strands (*13*, *18*–*20*) and ligands (*7*), and/or triggering interparticle interactions using external stimuli, such as chemical signaling (*21*–*24*) and physical fields (*25*–*32*). However, realizing self-assembly of dynamic colloidal structures along the vertical direction has been challenging because of gravity (*33*, *34*) and the complexity in controlling the interparticle bonds and interactions.

Here, we present a strategy that enables the self-assembly of gravity-resisting colloidal collectives, which are capable of adaptive locomotion and have swarm behavior. The self-assembly process is realized by the time-varying particle-particle interactions induced by a tailored alternating magnetic field. Upon energization by the field, particles assemble themselves against gravity into vertical collectives, and the structures grow until reaching dynamic equilibrium. The necessity of the vertical interparticle interactions, attraction among the intermediate structures, and positional reconfigurability of particles for the self-assembly of the vertical collectives are revealed. The collectives can perform adaptive locomotion with shape reconfiguration in different environments. Inspired by living swarms in nature, the collectives are endowed with swarm behavior to achieve gap and obstacle crossing and stair climbing. This work establishes a route for developing the next generation of smart materials and intelligent micromachines.

## RESULTS

### Self-assembly of vertical colloidal collectives

**Fig. 1.**
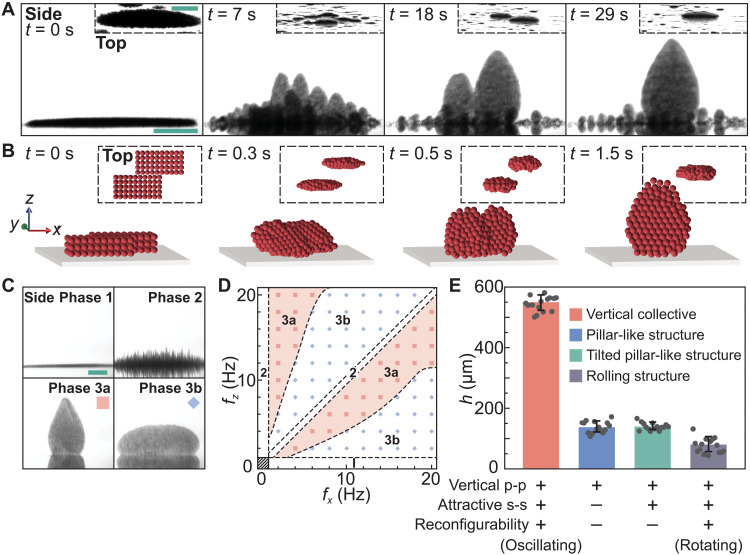
Generation of the colloidal collectives. (**A**) Dispersed particles dynamically assemble into a vertical collective when energized by the tailored magnetic field. Details are shown in fig. S5. (**B**) Simulation of collective generation. (**C**) Different phases of the colloidal structures generated by the tailored magnetic fields. The top views of the colloidal structures are shown in fig. S22. (**D**) Phase diagram showing the colloidal structures generated by the tailored magnetic fields with different combinations of the *x*-axial field frequency *f_x_* and *z*-axial field frequency *f_z_*. In (C) and (D), the corresponding phases of the colloidal structures are represented by 1, 2, and 3, respectively. The blue and red squares indicate that the collectives are primarily growing in horizontal and vertical directions, respectively. (**E**) The average heights *h* of the colloidal structures generated using different methods. The interactions between intermediate structures are indicated by s-s, while interparticle attraction is indicated by p-p. The reconfigurability here indicates the positional reconfigurability of the assembled particles. The legends indicate the patterns of the colloidal structures, and the patterns are shown in fig. S7. Each data point represents the height of a colloidal structure. The error bars represent the SDs. The “side” and “top” labels indicate the side view (projection onto the *xz* plane) and the top view (projection onto the *xy* plane), respectively. Scale bars, 100 μm.

The tailored field is uniquely composed of two sinusoidal oscillating magnetic fields with independently customized frequencies: one of which is parallel to the substrate, and the other of which is perpendicular to it (details in the “Applied magnetic field” section and fig. S1). A vertical collective is generated when the system is energized by the field (Fig. 1A and movie S1). The magnetic particles settled on the substrate assemble themselves against gravity to form small primitive collectives, that is, nonequilibrium growing collectives (at 7 s in Fig. 1A). The primitive collectives keep growing in height (before 18 s in Fig. 1A) and assemble with each other. Eventually, a vertical collective with a dynamic-equilibrium structure is generated and maintained (at 29 s in Fig. 1A).

The self-growing mechanism of the vertical collectives is revealed. By analyzing three main factors including the dipole-dipole interactions, gravity, and hydrodynamic drag, the collective generation process is simulated, as shown in Fig. 1B and movie S2 (details in the “Part A: Self-growing process of the pillars in the applied magnetic field,” “Part B: Oscillating behavior of the pillar in the applied magnetic field,” and “Part C: Generation of the vertical collective” sections and figs. S2 to S6). Driven by the tailored magnetic field, particles assemble themselves upward into oscillating pillar-like structures. Then, these pillars attract each other and form primitive collectives after a number of fragmentation and reformation cycles (before 0.5 s in Fig. 1B). Subsequently, the primitive collectives attract and merge with neighboring particles, contributing to their growth into a vertical collective (at 1.5 s in Fig. 1B). We elucidate that vertical interparticle interactions, attraction among the intermediate structures, and oscillating motions of particles are necessary in the self-assembly of vertical collectives. When vertical interparticle interactions are not induced, the particles cannot assemble upward (Fig. 1C, phase 1). When the vertical interparticle attraction is induced, the particles assemble into pillar-like structures (Fig. 1C, phase 2). These pillars experience repulsive interactions that hinder their assembly (*35*, *36*). The issue can be tackled by tilting the magnetic field, and in this case, the pillars incline and attract each other (details in the “Colloidal structures in different magnetic fields” section, fig. S7, and table S1) (*36*). However, the vertical growth of the pillars is limited because the assembled particles fail to reconfigure. The tailored dual-axis alternating magnetic field can thus be applied to actuate the oscillating motion of the pillars, resulting in structural fragmentation of the pillars, due to the balance of hydrodynamic (*37*, *38*), magnetic, and gravitational torques. As a result, the assembled particles are enabled to reconfigure into a vertical collective (Fig. 1C, phase 3a). The interparticle interaction can also be tuned to trigger the collective primarily growing along the horizontal direction (Fig. 1C, phase 3b), by tuning the frequencies of the tailored field (details in the “Part D: Growing direction of the collective” section and figs. S8 to S10). The phases of colloidal structures generated by the tailored magnetic fields with different combinations of frequencies are summarized in Fig. 1D, and the phase transitions are reversible (fig. S11). The colloidal structures generated in rotating magnetic fields are investigated. By applying a rotating magnetic field, the pillars break, and the particles reconfigure into rolling structures (fig. S7C) (*39*, *40*). However, these structures roll away from each other, and moreover, they generate streaming flows to repulse particles away, resulting in limited growth. The effectiveness of different methods in the generation of vertical structures is evaluated (Fig. 1E). The average heights of the vertical collectives are approximately 410 and 690% higher than that of the pillars and rolling structures, respectively, given the same experimental conditions.

### Gravity-resisting characteristics

**Fig. 2.**
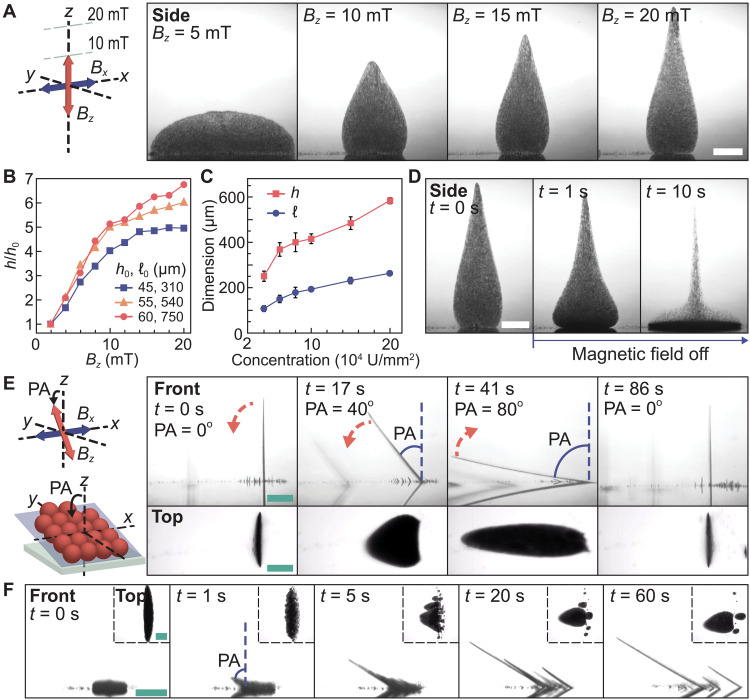
Gravity-resisting characteristics of the colloidal collectives. (**A**) Reconfiguration of a collective with growing height. (**B**) The changes in the height ratio of the collectives with the *z*-axial field strength *B_z_*. The height ratio is quantified by dividing the heights *h* of the collectives with their initial heights *h*_0_ measured at *B_z_* of 2 mT. The first and second values in the legends indicate the initial heights *h*_0_ and lengths ℓ_0_ of the collectives measured at *B_z_* of 2 mT, respectively. (**C**) Relationship between the collective dimension and initial areal particle concentration. The red and blue lines represent the average height *h* and length ℓ of the collectives. The magnetic field strength is kept constant at 20 mT. In (B) and (C), each data point represents the average of three measurements, and the error bars represent the SDs. (**D**) Collapse of a collective and falling of particles upon the removal of the magnetic field. (**E**) Inclination process of a collective. The schematics illustrate the tilted collective on a substrate. The blue region represents the oscillating plane of the collective. The red dashed arrows indicate the directions of inclination. PA is the pitch angle applied to the *z*-axial oscillating field *B_z_*. (**F**) Dispersed particles directly self-assemble into tilted collectives upon energized by the magnetic field with a PA of 60°. In (A) and (E), the schematics show the applied magnetic field with the blue and red arrows indicating the *x*-axial oscillating field *B_x_* and *z*-axial oscillating field *B_z_*, respectively. The side, top, and front labels indicate the side view (projection onto the *xz* plane), the top view (projection onto the *xy* plane), and the front view (projection onto the *yz* plane), respectively. Scale bars, 100 μm.

The gravity-resisting characteristics of the collectives are investigated. The shape of the collective is reconfigurable by tuning the magnetic field. By tuning the *z*-axial magnetic field strength *B_z_* from 5 to 20 mT, the height of the collective notably increases by 350% (from 144 to 504 μm in Fig. 2A). In this case, the height of the collective is equivalent to that stacked by 168 composing particles. The height reconfigurability of the collectives is evaluated through a height extension ratio, which is calculated by dividing the heights of the collectives with their initial heights. Depending on the sizes of the collectives, their heights can be extended up to approximately 700% (Fig. 2B), and they plateau when the number of composing particles is insufficient to support the vertical growth (blue line in Fig. 2B). We find that the reconfiguration capability of a collective is determined by the magnetic interactions among the composing pillars (details in the “Part E: Shape reconfigurability of the collective” section and fig. S12). When the *z*-axial magnetic field strength is increased, longer pillars form through magnetic assembly, and the vertical magnetic attractive forces lead to a higher height of the collective resisting gravity. In contrast, the height of the collective becomes lower when the *z*-axial magnetic field strength is decreased because the interpillar interaction is weakened, and gravity pulls the pillars downward. Other than tuning the magnetic field strength, the average heights of the collectives can also be increased by raising the areal concentration of particles (Fig. 2C). By reconfiguring the height and length of the collectives, we show that they can be merged to form a larger collective (see the “Controlled merging of the collectives” section, fig. S13, and movie S1). Through the merging process, the collective with the height of approximately 725 μm, equivalent to the height stacked by 242 composing particles, can be achieved (fig. S14).

We further investigate the gravity-resisting characteristics of the collectives. On the removal of the magnetic field, the collective collapses and the particles fall instantaneously, validating that the particles are influenced notably by gravity (Fig. 2D and movie S1). Despite the influence of gravity, the collective can be tilted with a large angle toward the substrate, such as 80°, resisting gravity (Fig. 2E and movie S1). During the tilting process, the collective’s integrity is maintained and no falling of particles occurs, indicating that the magnetic interactions among the particles are sufficiently strong to counterbalance gravity even when the collective is tilted (details in the “Part F: Inclination of the collective” section and fig. S15). The tilted collective can be reversed to the vertical configuration. Moreover, the particles can also resist gravity to directly assemble into tilted collectives using the proposed strategy, without forming an equilibrium collective beforehand. By applying the tailored magnetic field with a 60° pitch angle, a tilted collective is generated directly from dispersed particles (Fig. 2F, movie S1, and fig. S16).

### Hydrodynamic characteristics and adaptive locomotion

**Fig. 3.**
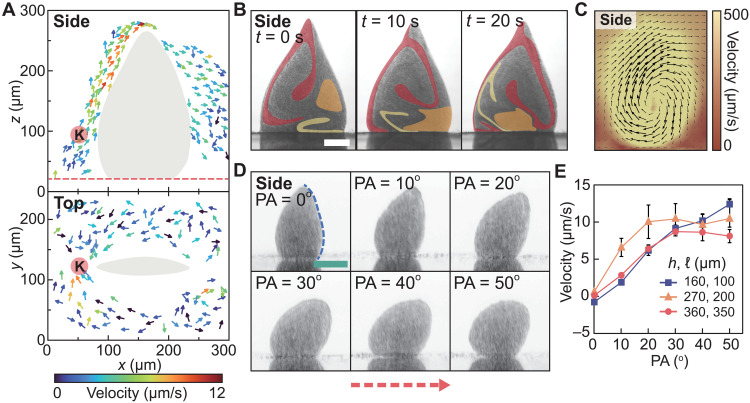
Hydrodynamic and motion characteristics of the colloidal collectives. (**A**) The tracked motion of polystyrene tracing particles. The gray regions outline the collective. The colored arrows indicate the moving directions of the tracers, and the colors indicate the velocities of the tracers. The red spots K indicate the corresponding places in the side view and the top view. (**B**) Swirling particles in a collective. The red, orange, and yellow regions indicate the distribution of three different groups of particles. (**C**) Simulation of the hydrodynamic vortex induced by the motions of the composing particles of a collective. The background colors indicate the velocities of the flow field. (**D**) Structural changes of a collective with the pitch angle of the applied magnetic field. PA is the pitch angle applied to the *x*-axial oscillating field *B_x_*. The red dashed arrows indicate the moving direction of the collective. The blue dashed line indicates the frontal area of the collective. (**E**) The changes in the velocities of the collectives with the pitch angle of the tailored magnetic field. The first and second values in the legends indicate the heights and lengths of the collectives, respectively. Each data point represents the average of three repeated measurements. The error bars represent the SDs. The side and top labels indicate the side view (projection onto the *xz* plane) and the top view (projection onto the *xy* plane), respectively. Scale bars, 100 μm.

Streaming flow field is generated by the collective (Fig. 3A). The flow is profiled by deploying and tracking polystyrene tracing particles. As the side view shows, the tracers move upward along the left side of the collective to the peak with increasing translational velocities, and then they move away from the collective on the other side. From the top view, the tracers converge to red spot “K”; from the side view, spot K locates on the left side of the collective. Therefore, the motions of the tracers reveal that a streaming flow is generated, converging toward the left side of the collective and then rising toward the peak of the collective. The streaming flow is induced by the swirling motions of the particles inside the collective, due to the asymmetries in the directions of the time-varying magnetic fields (details in the “Part G: Asymmetrical oscillation of the collective” section and fig. S17). Three groups of particles are traced with colors as they move in a spiral pattern (Fig. 3B). Simulation results show that hydrodynamic vortexes are induced by the particles (Fig. 3C and fig. S17) and the asymmetrical velocity distribution of the vortexes leads to the generation of the streaming flow.

**Fig. 4.**
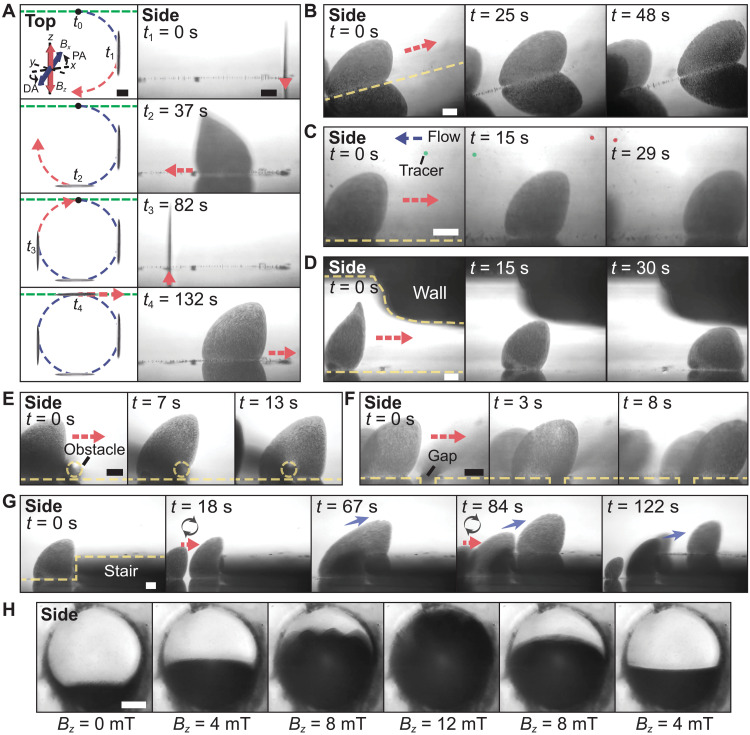
Adaptive locomotion and swarm behavior of the colloidal collectives. (**A**) A collective is navigated to perform locomotion along a circular path. The schematics show the applied magnetic field with the blue and red arrows indicating the *x*-axial oscillating field *B_x_* and the *z*-axial oscillating field *B_z_*, respectively. PA and DA are the pitch angle and the direction angle applied to the *x*-axial oscillating field *B_x_*, respectively. The black dots indicate the initial position of the collective, and the blue dashed lines represent the trajectory of the collective. The green dashed lines indicate the focal plane of the side microscope. (**B**) A collective performs locomotion along a slope with a 15° tilted angle. (**C**) A collective moves against fluidic flow (approximately 50 μm/s). (**D**) A collective is reconfigured to adapt into and pass through a confined channel. (**E**) A collective crosses an obstacle. The obstacle is a copper wire with a diameter of 80 μm, which is outlined by the yellow dashed circles. (**F**) A collective crosses a gap with a width of 80 μm. (**G**) The collectives work together to climb up a stair with a height of 200 μm. (**H**) The collectives work together to cover the light path in a circular microchannel with a diameter of 400 μm. In (A) to (E), the red dashed arrows indicate the moving directions of the collectives. In (G), the red arrows indicate the moving directions of the incoming collectives, while the blue arrows indicate the moving directions of the departing collectives. The black arrows indicate the merging of the collectives. The side and top labels indicate the side view (projection onto the *xz* plane) and the top view (projection onto the *xy* plane), respectively. Scale bars, 100 μm.

For micromachines, to enhance their mobility is a major goal (*41*). The collectives can serve as micromachines with the capabilities of locomoting themselves and performing on-demand shape reconfiguration. The translational motion of the collective is associated with the symmetry breaking of the collective oscillation (details in the “Part G: Asymmetrical oscillation of the collective” section and fig. S18). By tuning the pitch angle of the *x*-axial magnetic field *B_x_*, the collective reconfigures (Fig. 3D) and moves forward. The collectives move faster when the pitch angle increases (Fig. 3E) because their frontal areas (blue dashed line in Fig. 3D) decrease and, hence, their experienced hydrodynamic drags also decrease. When the pitch angle exceeds 30°, the contact areas between the collectives and the ground decrease (Fig. 3D), which reduce the translational distance per oscillating cycle, and, hence, the velocities of the collectives decrease (orange and red lines in Fig. 3E). The velocities of the collectives can be increased by increasing the magnetic field strength (fig. S19). Moreover, the orientation of the collective can be controlled by tuning the direction of the magnetic field (fig. S20). We then show that the collective can be navigated to follow a circular path (Fig. 4A and movie S3). From 0 to 37 s, the collective is navigated out of the side microscope’s focal plane that is indicated by the green dashed lines in the top views. At 132 s, the collective completes the circular path and is back to the focal plane. The locomotion capability of the collectives in different complex environments is testified (movie S3). A collective can move along a slope with a 15° tilted angle (Fig. 4B), indicating that the interparticle magnetic interactions are capable of pulling the particles upward and forward. Besides the vertical interactions, horizontal confining forces are also induced among particles to maintain the integrity of the collectives against external disturbances. As shown in Fig. 4C, the collective can move against hydrodynamic flow (approximately 50 μm/s) without sacrificing its intactness. In this case, the *x*-axial components of the interparticle interactions counterbalance the hydrodynamic drag forces. When the collectives encounter an environment with height confinements, they are able to perform on-demand shape reconfiguration to adapt to the confined space. By tuning the *z*-axial magnetic field strength, the height of a collective is lowered to fit into and pass through a confined channel with a low ceiling (Fig. 4D and movie S3).

### Swarm behavior

Inspired by living swarm intelligence in nature, such as fire ants cooperate to keep their colonies in response to disasters (*42*), the collectives have swarm behavior mimicking the ants, relying on the programmable interparticle interactions. The collectives are capable of crossing an obstacle and a gap (Fig. 4, E and F, and movie S4), due to the collaboration among the composing particles. When a collective encounters an obstacle, the particles in its upper part move beyond the obstacle first and then pull the bottom particles upward to cross the obstacle, using the *z*-axial magnetic interactions (Fig. 4E). Meanwhile, to guarantee the collective to cross a gap with an intact shape, the *z*-axial magnetic interactions provided by the upper particles of the collective also play a critical role in keeping the lower particles in place (Fig. 4F). Moreover, the collectives are capable of cooperating to climb up a high stair (Fig. 4G and movie S4). When a small collective reaches a stair, because of the size limitation, it stays under the stair, merges with more arriving collectives, and grows. As a result, the bottom part of the collective keeps serving as a ladder for the arriving collectives to climb up the stair. Relying on this similar swarm behavior, the collectives can also pass through a wide gap beyond their sizes (fig. S21 and movie S4). First arriving collectives drop into the gap and serve as a bridge for subsequently arriving collectives. Using the reconfiguration capability, the collectives can cooperate, for instance, to act as a tunable optical aperture in a circular microchannel. The collectives gradually grow to block the light path in the channel when the *z*-axial magnetic field strength is increased from 0 to 12 mT (Fig. 4H and movie S4).

## DISCUSSION

In this work, we reveal the necessity of the vertical interparticle interactions, attraction among the intermediate structures, and positional reconfigurability of particles for the self-growing of gravity-resisting dynamic collectives. The strategy that we proposed provides a prototypical paradigm for triggering the self-assembly of dynamic colloidal structures along the vertical direction. Counterbalancing the gravitational force, the interparticle magnetic interactions induced by the tailored magnetic field drive the particles to self-assemble into vertical collectives. The shape, orientation, inclination, and locomotion of the collectives can be controlled on-demand via tuning the parameters of the magnetic field. Programmable interactions among the particles give rise to swarm behavior of the collectives, with the capability of mimicking ant colonies in overcoming complex environments. The proposed strategy can potentially be integrated with other forms of physical interactions for advancing colloidal self-assembly toward the creation of functional micro/nanosystems.

## MATERIALS AND METHODS

### Materials and setup for experiments

Paramagnetic particles with an average diameter of 3 μm (ProMag HP, commercially available on Bangs Laboratories Inc.) are used in the experiments. The density of the particles is 1.4 g/cm^3^, and the surface is hydrophilic. The particles are suspended in 0.1% Tween 20 solution to prevent nonspecific aggregations. A transparent glass tank is used to integrate with side-view optical microscopic imaging (fig. S23). Two silicon wafers are installed at the bottom and side of the tank with their polished surface upward (facing the top light source) and inward (facing the side light source), respectively, to enhance the observation contrast. The glass tank is fully filled with water-based solution (~2 ml). No difference in self-assembly is observed in water-based salt solution (phosphate-buffered saline, Gibco) and deionized water (Sigma-Aldrich). One drop of the particle solution (1 μl, 0.02 weight %) is added into the tank (~4 mm above the substrate) and is left for particles to settle on the substrate (~5 min). Magnetic actuation is conducted in a three-axis Helmholtz electromagnetic coil setup (fig. S23). The actuation signals are generated by a computer, and then the current is input into the coils to generate magnetic fields in the working space. MATLAB and ImageJ are used for analysis.

### Applied magnetic field

The magnetic field consists of two oscillating magnetic fields, with one *x*-axial field *B_x_*(*t*) = *A* sin (2π*f_x_t*) and one *z*-axial field *B_z_*(*t*) = γ*A* sin (2π*f_z_t*), as shown in fig. S1A. The resultant magnetic field **B** can be expressed as

$$\;B(t)={\;B}_{\;x}(t)+{\;B}_{\;z}(t)=A\sin(2\pi {\;f}_{x}t){\overset{⌢}{e}}_{\;x}+\gamma A\sin(2\pi {\;f}_{z}t){\overset{⌢}{e}}_{\;z}$$ (1)

where *A* is the amplitude of the oscillating fields, γ is an amplitude ratio (γ = *B_z_*/B*_x_*), and *t* is time. The frequency of the *x*-axial oscillating field *B_x_* and that of the *z*-axial oscillating field *B_z_* are represented by *f_x_* and *f_z_*, respectively. The oscillating angle ϕ(*t*) and angular velocity **ω**(*t*) of the resultant magnetic field **B** can be expressed as

$$\varphi (t)=\arctan\frac{\gamma \sin(2\pi {\;f}_{z}t)}{\sin(2\pi {\;f}_{x}t)}$$ (2)

$$\text{ω}(t)=\frac{d\varphi (t)}{dt}{\overset{⌢}{e}}_{\;y}=\frac{-4\pi^{2}\gamma {\;f}_{x}{\;f}_{z}\cos(2\pi {\;f}_{x}t)\cos(2\pi {\;f}_{z}t)}{\sin^{2}(2\pi {\;f}_{x}t)+\gamma^{2}\sin^{2}(2\pi {\;f}_{z}t)}{\overset{⌢}{e}}_{\;y}$$ (3)

The profiles of the resultant magnetic field **B** with a field strength *A* of 10 mT, an amplitude ratio γ of 1, an *x*-axial field frequency *f_x_* of 1 Hz, and a *z*-axial field frequency *f_z_* of 10 Hz are plotted (fig. S1, B and C). The change in the field oscillating angle ϕ and that in the field angular velocity **ω** with time are also plotted (fig. S1D).

### Analysis

#### Part A: Self-growing process of the pillars in the applied magnetic field

Upon energized by the magnetic field, dispersed particles interact with each other and assemble into pillar-like structures. The forces experienced by a particle are analyzed. The paramagnetic particles are assumed to have the same diameter *D*, density ρ*_r_*, volume *V_r_*, and effective magnetic susceptibility χ*_r_*. The forces exerted on particle *i*, including the magnetic dipole-dipole interaction force (*43*) ${\;F}_{\;i}^{\;m}$, hydrodynamic drag force ${\;F}_{\;i}^{\;d}$, and gravitational force **F**_**g**_ can be expressed as

$${\;F}_{\;i}^{\;m}=\frac{3\mu_{0}}{4\pi }\sum_{i\neq j,j=1}^{N}\frac{m{{}_{i}m}_{j}}{r_{ij}^{4}}({\overset{⌢}{r}}_{\mathrm{ij}}({\overset{⌢}{m}}_{\;i}\cdot {\overset{⌢}{m}}_{\;\;j})+{\overset{⌢}{m}}_{\;i}({\overset{⌢}{r}}_{\mathrm{ij}}\cdot {\overset{⌢}{m}}_{\;\;j})+{\overset{⌢}{m}}_{\;\;j}({\overset{⌢}{r}}_{\mathrm{ij}}\cdot {\overset{⌢}{m}}_{\;i})-5{\overset{⌢}{r}}_{\mathrm{ij}}({\overset{⌢}{r}}_{\mathrm{ij}}\cdot {\overset{⌢}{m}}_{\;i})({\overset{⌢}{r}}_{\mathrm{ij}}\cdot {\overset{⌢}{m}}_{\;\;j}))$$ (4)

$${\;F}_{\;i}^{\;d}=-C_{i}({\dot{\;r}}_{\;i}-{\;v}_{\;\;f})$$ (5)

$${\;F}_{\;g}=-(\rho_{r}-\rho_{f})V_{r}g{\overset{⌢}{e}}_{\;z}$$ (6)

where μ_0_ = 4π × 10^−7^ H/m is the permeability of free space, *N* is the number of particles, *j* indicates the *j*th surrounding particles, $\;m=\frac{V_{r}\chi_{r}\;B}{\mu_{0}}$ is the magnetic moment of the particle, **B** is the vector of the magnetic field, *r_ij_* = ∣**r**_**j**_ − **r**_**i**_∣ is the distance between the center of mass of particle *i* and that of particle *j*, *C* = 3πη*D* is the effective Stokes’ drag coefficient for a free spherical particle, η is the dynamic viscosity of the fluid, $\dot{\;r}$ is the velocity of a particle, **v**_**f**_ is the flow field of the background fluid, *g* = ∣**g**∣ is the amplitude of the gravity field, and ρ*_f_* is the density of the fluid. The magnetic moments of the particles are assumed to be the same, that is, **m**_**i**_**=m**_**j**_**=m**. Therefore, the magnetic dipole-dipole interaction force ${\;F}_{\;i}^{\;m}$ can be simplified to

$${\;F}_{\;i}^{\;m}=B^{2}M\sum_{i\neq j,j=1}^{N}\frac{1}{r_{ij}^{4}}{\overset{⌢}{R}}_{\mathrm{ij}}$$ (7)

where $M=\frac{3{V_{r}}^{2}{\chi_{r}}^{2}}{4{\pi \mu }_{0}}$ characterizes the magnetic properties of the particles, *B* = ∣**B**∣ is the magnetic field strength, and ${\overset{⌢}{R}}_{\mathrm{ij}}=(1-5{\left(\hat{\;B}\cdot {\hat{\;r}}_{\mathrm{ij}}\right)}^{2}){\hat{\;r}}_{\mathrm{ij}}+2(\hat{\;B}\cdot {\hat{\;r}}_{\mathrm{ij}})\hat{\;B}$. From Eq. 7, the magnetic dipole-dipole interaction force ${\;F}_{\;i}^{\;m}$ experienced by a particle increases when the magnetic field strength *B* increases.

To model solid spherical particles, the Lennard-Jones interaction force is implemented as the particle-particle excluded volume force ${\;F}_{\;i}^{\mathrm{ev}}$ (*37*), which can be expressed

$${\;F}_{\;i}^{\mathrm{ev}}=\frac{24\varepsilon {}_{r}}{\sigma_{r}}\sum_{i\neq 1,j=1}^{N}(2{\left(\frac{\sigma_{r}}{r_{ij}}\right)}^{13}-{\left(\frac{\sigma_{r}}{r_{ij}}\right)}^{7})\;{\overset{⌢}{r}}_{\mathrm{ij}}$$ (8)

where ε*_r_* is the interparticle interaction strength and σ*_r_* is the interparticle collision diameter.

The repulsive force between the wall and the particle, ${\;F}_{\;i}^{\mathrm{wp}}$, acting along the *z* axis, is approximated using modified Lennard-Jones potential with a corresponding force

$${\;F}_{\;i}^{\mathrm{wp}}=\frac{24\varepsilon_{w}}{\sigma_{w}}(2{\left(\frac{\sigma {}_{w}}{r_{z}-a}\right)}^{12}-{\left(\frac{\sigma_{w}}{r_{z}-a}\right)}^{6})\;\overset{⌢}{z}$$ (9)

where ε*_w_* is the interaction strength between the wall and particle, σ*_w_* is the collision diameter between the wall and the particle, *r_z_* is the distance from the wall to the center of the particle, and *a* is the radius of the particle.

The movement of particles through the fluid will influence the corresponding flow field **v**_**f**_, and, consequently, the movements of the other particles are affected. Hydrodynamic interactions among the particles are analyzed by considering the stokeslet of spherical particles. The volume force **F**_**v**_ exerted on the fluid at position **r**_**j**_ by particle *j* is equal in magnitude but opposite in direction to the hydrodynamic drag force ${\;F}_{\;\;j}^{\;d}$experienced by particle *j*, that is, ${\;F}_{\;v}({\;r}_{\;\;j})=-{\;F}_{\;\;j}^{\;d}$. In Stokes flow, a linear relationship exists between the volume force **F**_**v**_(**r**_**j**_) and the velocity perturbation of the flow field **v**_**f**_(**r**_**k**_) at the other position **r**_**k**_. The total velocity perturbation of the flow field at position **r**_**k**_ caused by *N* number of particles in unbound fluid can be expressed (*37*).

$${\;v}_{\;\;f}({\;r}_{\;k})=\sum_{\;j=1,j\neq k}^{N}\;G({\;r}_{\mathrm{kj}})\;{\;F}_{\;v}({\;r}_{\;\;j})$$ (10)

where $\;G({\;r}_{\mathrm{kj}})=\frac{1}{8\text{π}\text{η}r_{kj}}({\;I}_{\mathrm{kj}}-{\hat{\;r}}_{\mathrm{kj}}{\hat{\;r}}_{\mathrm{kj}})$ is the Oseen tensor, **I**_**kj**_ is the identity tensor, and **r**_**kj**_ is the vector from a point force at position **r**_**j**_ to position **r**_**k**_ with the magnitude *r_kj_* and direction unit vector ${\hat{\;r}}_{\mathrm{kj}}$. In the presence of a substrate, the velocity at the surface is zero, that is, *v_f_*(*z* = 0) = 0.

From Eqs. 1 and 5 to 10, the velocity of particle *i* can be expressed as

$${\dot{\;r}}_{i}=\frac{1}{C_{i}}({\;F}_{\;i}^{\;m}+{\;F}_{\;g}+{\;F}_{\;i}^{\mathrm{ev}}+{\;F}_{\;i}^{\mathrm{wp}})+{\;v}_{\;\;f}$$ (11)

From Eq. 11, the velocities of the particles are influenced by the gravity, surrounding flow, and time-varying field-induced magnetic interactions.

The behaviors between a particle and a pillar are simulated and investigated, as shown in fig. S2. The field strength *A*, amplitude ratio γ, *x*-axial field frequency *f_x_*, and *z*-axial field frequency *f_z_* of the applied magnetic field are 10 mT, 1, 1 Hz, and 10 Hz, respectively. The blue arrows represent the vectors of the magnetic field. The composing particles of the pillar are in red, and the single particle is in blue. Upon energized, the blue particle interacts with the pillar. From 0 to 0.04 s, the interaction is weak, and the blue particle remains stationary. The pillar breaks when the magnetic field oscillates with a relatively higher angular velocity, as shown from 0.04 to 0.06 s. From 0.08 to 0.13 s, the fragment pulls the blue particle upward, and, eventually, the blue particle merges into the lower part of the pillar and contributes to the pillar growth.

#### Part B: Oscillating behavior of the pillar in the applied magnetic field

The pillar oscillates with the input oscillating magnetic field. The oscillating motion of the pillar is led by the tangential components of the magnetic dipole-dipole interaction forces, which can be expressed as

$${\;F}_{\;i}^{\theta }=B^{2}M\sin(2\alpha )\sum_{i\neq j,j=1}^{N}\frac{1}{r_{ij}^{4}}\;{\overset{⌢}{e}}_{\theta }$$ (12)

where $M=\frac{3{V_{r}}^{2}{\chi_{r}}^{2}}{4{\pi \mu }_{0}}.$ characterizes the magnetic properties of the particles, *V_r_* is the volume of the particles, χ*_r_* is the effective magnetic susceptibility of the particles, μ_0_ is the permeability of free space, *B* is the magnetic field strength, α is the phase lag angle between the magnetic field and the long axis of the pillar, *N* is the number of particles in the pillar, and *r_ij_* is the distance between the center of mass of particle *i* and that of particle *j*.

During the oscillating motion, the driven magnetic torque **Γ**_**m**_ and resistive viscous torque **Γ**_**v**_ are exerted on the pillar, as shown in fig. S3A. The driven magnetic torque **Γ**_**m**_ is determined by the ${\;F}_{\;i}^{\theta }$ exerted on the outer particles of a pillar (*38*), and we assume that α = π/4. In this case, the driven magnetic torque **Γ**_**m**_ is the greatest, which can be expressed as

$$\Gamma_{\;m}=B^{2}M\frac{1}{D^{3}}(N-1){\overset{⌢}{e}}_{\;y}$$ (13)

where *D* is the diameter of the particles.

The viscous torque (*38*) **Γ**_**v**_ exerted on the pillar can be expressed as

$$\Gamma_{\;v}=\frac{1}{3}\pi D^{3}\eta \text{ω}\frac{N^{3}}{\left(\ln(N/2)+2.4/N\right)}$$ (14)

where η is the dynamic viscosity of the fluid and **ω** is the angular velocity of the pillar.

Moreover, when the magnetic field is removed, the collective collapses and the particles fall because of gravity (Fig. 2D), indicating that the influence of gravity is notable in the system and may contribute to the oscillating behavior of the pillar. The gravitational torque **Γ**_**g**_ exerted on the pillar can be expressed as

$$\Gamma_{\;g}=\frac{1}{12}(\rho_{r}-\rho_{f})g\pi D^{4}\cos(\kappa )N^{2}{\overset{⌢}{e}}_{\;y}$$ (15)

where ρ*_r_* is the density of the particles, ρ*_f_* is the density of the fluid, *g* = ∣**g**∣ is the amplitude of the gravity field, and κ is the oscillating angle between the long axis of the pillar and the substrate.

The opposing torques are dependent on the angular velocity of the magnetic field ω and the oscillating angle of the pillar κ, which can be expressed as

$$\Gamma_{v}=\left\{ \begin{array}{ll} \Gamma_{m}-\Gamma_{g}, & \;\left(0<\kappa <\pi /2\cap \omega >0)\cup (\pi /2<\kappa <\pi \cap \omega <0\right)\\ \Gamma_{m}, & \;\kappa =\pi /2\\ \Gamma_{m}+\Gamma_{g}, & \left(0<\kappa <\pi /2\cap \omega <0)\cup (\pi /2<\kappa <\pi \cap \omega >0\right) \end{array}\right.$$ (16)

From Eq. 16, the number of particles in a pillar is the lowest when the driven magnetic torque **Γ*_m_*** is counterbalanced by the viscous torque **Γ*_v_*** and gravitational torque **Γ*_g_***.

The dimensionless Mason number is used to characterize the dynamics of a rotating or an oscillating chain (*38*, *44*). Meanwhile, the fragmentations of the chains are notably influenced by the number of composing particles and the angular velocity of the chain (*37*, *38*). Gravity plays a role in the collective generation. Therefore, a modified Mason number *R_T_* including the gravitational torque is derived, which can be expressed as

$$R_{T}=\frac{\Gamma_{g}+\Gamma_{v}}{\Gamma_{m}}=\frac{4\mu_{0}g(\rho_{r}-\rho_{f})D\cos(\kappa )}{B^{2}{\chi_{r}}^{2}}\frac{N^{2}}{N-1}+\frac{16\mu_{0}\eta \omega }{B^{2}{\chi_{r}}^{2}}\frac{N^{3}}{\left(N-1)(\ln(N/2)+2.4/N\right)}$$ (17)

The modified Mason number *R_T_* is derived on the basis of the situation when the driven magnetic torque **Γ*_m_*** is counterbalanced by the viscous torque **Γ*_v_*** and gravitational torque **Γ*_g_***, in which the pillar will be the shortest. When the modified Mason number *R_T_* is smaller than unity, it indicates that a pillar oscillates without fragmentation. From Eq. 17, when the angular velocity of the magnetic field ω is considerably low, that is, the viscous torque **Γ*_v_*** is negligible, a pillar grows until the driven magnetic torque **Γ*_m_*** is counterbalanced by the gravitational torque **Γ*_g_***. In contrast, when the angular velocity of the magnetic field ω is high, the pillar undergoes fragmentation if it grows beyond the corresponding number of composing particles *N*. The fragmentation of a pillar is expected to occur regularly because of the periodical changes in the angular velocity of the magnetic field (fig. S1D).

The simulated behavior of an oscillating pillar in the magnetic field is investigated, as shown in fig. S3B. The field strength *A*, amplitude ratio γ, *x*-axial field frequency *f_x_*, and *z*-axial field frequency *f_z_* of the applied magnetic field are 10 mT, 1, 1 Hz, and 10 Hz, respectively. The blue arrows represent the vectors of the magnetic field. The oscillating pillar undergoes fragmentation and reformation. From 0.175 to 0.225 s, the pillar breaks into three fragments (two fragments with one particle and one fragment with four composing particles). In the following 0.05 s, the longer fragments further break into two fragments (each with two composing particles), and then the reformation of two pillars occurs (at 0.275 s) because the fragments attract each other. When the angular velocity of the field is relatively low, from 0.375 to 0.5 s, all the fragments attract each other, and the pillar is reformed.

#### Part C: Generation of the vertical collective

The self-growing mechanism of the vertical collective is investigated, as illustrated in fig. S4. In the oscillating magnetic field, dispersed particles assemble themselves to form pillars (at *t*_1_), and the pillars grow upward through merging (at *t*_2_). These growing pillars attract each other through magnetic interactions, and a primitive collective is formed (at *t*_3_), that is, a nonequilibrium growing collective. The core part of the primitive collective (labeled by the black boxes) performs rigid-body oscillation, while the pillars near the edge undergo fragmentation (at *t*_4_). The fragments attract each other and form the upper part of the collective (at *t*_5_). The lower part of the collective continuously attracts and merges with the surrounding particles, contributing to the growth of the vertical collective (at *t*_6_).

The experimental generation of the vertical collective is investigated, as shown in fig. S5. The field strength *A*, amplitude ratio γ, *x*-axial field frequency *f_x_*, and *z*-axial field frequency *f_z_* of the applied magnetic field are 10 mT, 1, 1 Hz, and 10 Hz, respectively. Dispersed particles form primitive collectives from 0 to 2 s. These primitive collectives grow and merge with each other. At 8 s, vertical collectives are generated. Repulsion is induced between the sides of the collectives due to the repulsive magnetic interactions induced between the pillars in one collective and those in the other collective. Therefore, the collectives merge with each other from their fronts (refer to fig. S4B), from 8 to 24 s. Eventually, a vertical collective is generated at 26 s.

The number of particles required for collective generation is investigated, as shown in fig. S6. The field strength *A*, amplitude ratio γ, *x*-axial field frequency *f_x_*, and *z*-axial field frequency *f_z_* of the applied magnetic field are 10 mT, 1, 1 Hz, and 10 Hz, respectively. The height of the residual pillars is within 16 μm, which consist of approximately eight particles, as shown in fig. S6B. Using the modified Mason number in Eq. 17, fragmentation of a pillar occurs when the number of composing particles exceeds 12, given the applied magnetic field condition and the measured average angular velocity of ~3.14 rad/s. Therefore, it can be expected that the residual pillars remain in the pillar form if they cannot merge with more particles. The number of particles required for collective generation is experimentally determined by measuring the dimension of the smallest collective in 10 trials. The average dimension of the smallest collective (fig. S6B) is measured to be 16 μm in height, 14 μm in length, and 9 μm in width, consisting of approximately 75 particles.

#### Part D: Growing direction of the collective

The collectives have higher tendencies growing in one direction than the other (either vertical or horizontal direction) when the dual-axis oscillating fields with different combinations of frequencies are applied. These oscillating fields can be categorized into two types: longitudinal fields and lateral fields (fig. S8A). In longitudinal fields, the vectors of the magnetic fields oscillate upward and downward regularly, while they keep oscillating from side to side in lateral fields. The particles form pillars with different oscillating behaviors in these two types of fields (fig. S8B), assuming that there is no phase lag between the oscillations of the fields and those of the particles. Driven by the longitudinal field, a pillar oscillates downward aligning parallel to the substrate (from *t*_1_ to *t*_2_ and *t*_4_ to *t*_5_) and then oscillates upward (at *t*_3_ and *t*_6_). In the lateral field, the pillar aligns perpendicular to the substrate (at *t*_2_ and *t*_5_) during its side-to-side oscillation.

The simulated behaviors of the pillars in the longitudinal field and the lateral field are shown in fig. S9. The pillars perform up-down and side-to-side oscillations in the longitudinal and lateral fields, respectively. When the pillars oscillate, they break because of viscous torque (from 0.14 to 0.19 s, 0.39 to 0.44 s, 0.64 to 0.69 s, and 0.89 to 0.94 s) and fall under gravity. The up-down oscillating fragments attract each other to reform pillars along the horizontal direction (at 0.39, 0.64, and 0.89 s of fig. S9A), while the side-to-side oscillating fragments reform pillars along the vertical direction (at 0.39, 0.64, and 0.89 s of fig. S9B). After a number of fragmentation and reformation, for the case where pillars exhibit up-down oscillations, the number of the particles along the horizontal direction is higher than that along the vertical direction (fig. S9A), which is the feature observed in phase 3b, as shown in Fig. 1C. In contrast, the side-to-side oscillating pillars self-assemble vertically with the number of the particles along the vertical direction being higher than that along the horizontal direction, entering phase 3a (Fig. 1C).

The experimental oscillation and growth of the vertical collectives in the longitudinal and lateral oscillating fields are shown in fig. S10 and movie S5. When the frequencies of the oscillating fields are low (i.e., 4 Hz), the oscillations of the collectives are in-phase with those of the fields. In contrast, when the field frequencies are high (i.e., 10 Hz), the collectives enter step-out states, and their oscillations are opposite in direction to that of the fields. In the cases of low-frequency lateral and high-frequency longitudinal oscillating fields, the collectives show side-to-side oscillations and primarily grow in the vertical direction (fig. S10, A and D). As supported by the simulation, side-to-side oscillating pillars self-assemble vertically during their fragmentation and reformation process (fig. S9B). This vertically growing phase is denoted by phase 3a in Fig. 1D. In the high-frequency lateral and low-frequency longitudinal oscillating fields, the collectives show up-down oscillations (fig. S10, B and C). Because the interparticle interactions lead the particles to align horizontally (fig. S9A), the collectives primarily grow in the horizontal direction. This horizontally growing phase is denoted by phase 3b in Fig. 1D.

#### Part E: Shape reconfigurability of the collective

To better understand the reconfiguration mechanism of the collective, the structural change of a pillar in response to the *z*-axial field strength component *B_z_* is investigated. The modified Mason number *R_T_* in Eq. 17 is inversely proportional to the square of the magnetic field strength. It indicates that if the *z*-axial field strength component *B_z_* decreases, then a pillar could be broken into shorter fragments. The magnetic interaction forces between the fragments **F**^**p**^ can be calculated by summing the magnetic dipole-dipole interaction forces ${\;F}_{\;i}^{\;m}$ exerted on each particle of a fragment, which can be expressed as

$${\;F}^{\;\;p}=B^{2}M\sum_{i\neq j,j=1}^{N_{\mathrm{total}}}\frac{1}{r_{ij}^{4}}{\overset{⌢}{R}}_{\mathrm{ij}}$$ (18)

where *N*_total_ is the total number of particles in the fragments, $M=\frac{3{V_{r}}^{2}{\chi_{r}}^{2}}{4{\pi \mu }_{0}}$ characterizes the magnetic properties of the particles, *V_r_* is the volume of the particles, χ*_r_* is the effective magnetic susceptibility of the particles, μ_0_ is the permeability of free space, *B* is the magnetic field strength, *r_ij_* is the distance between the center of mass of particle *i* and that of particle *j*, and ${\overset{⌢}{R}}_{\mathrm{ij}}=(1-5{\left(\hat{\;B}\cdot {\hat{\;r}}_{\mathrm{ij}}\right)}^{2}){\hat{\;r}}_{\mathrm{ij}}+2(\hat{\;B}\cdot {\hat{\;r}}_{\mathrm{ij}})\hat{\;B}$.

From Eq. 18, the magnetic interaction forces between the fragments **F**^**p**^are weaker when the magnetic field strength *B* or the total number of particles in the fragments *N*_total_ is lower. Therefore, when the *z*-axial field strength component *B_z_* is lower, shorter fragments are formed (according to Eq. 17), and their magnetic interaction forces **F**^**p**^ are weaker (according to Eq. 18).

The simulated behavior of a pillar in the field with *z*-axial strength *B_z_* of 2 mT and that of 20 mT is investigated, respectively, as shown in fig. S12. Initially, the field strength *A*, amplitude ratio γ, *x*-axial field frequency *f_x_*, and *z*-axial field frequency *f_z_* of the applied magnetic field are 10 mT, 1, 1 Hz, and 10 Hz, respectively. When the *z*-axial strength of the field *B_z_* is tuned to 2 mT, the pillar (with eight composing particles) breaks into eight short fragments (each with one particle). These fragments fall because of gravity, resulting in a shorter pillar. When the *z*-axial strength of the field *B_z_* is tuned to 20 mT, the fragments reform a pillar (with eight composing particles). The simulation provides insight into the reconfiguration mechanism of the collective, which is driven by the resultant interaction of the gravity and magnetic interactions.

#### Part F: Inclination of the collective

When a pitch angle is applied to the *z*-axial oscillating magnetic field, the direction of the magnetic field vector changes. As the direction of the field vector is different from that of the magnetic dipole moment of the particles, magnetic torques (Eq. 13) are exerted on the particles aligning them parallel to the field vector. As a result, the collective is tilted (fig. S15). Inside the collective, the magnetic dipole-dipole interaction forces **F**^**m**^ exerted on a particle (by the neighboring particles) counterbalance each other when the particle configuration remains unchanged (fig. S15B, inset). However, along the *z* axis, the particle experiences the gravitational force **F**^**g**^ (~0.1 pN), which pulls it down and affects the forces **F**^**m**^. To retain the particle inside the collective, the gravitational force **F**^**g**^ needs to be counterbalanced by the *z*-axial component of the resultant magnetic dipole-dipole interaction forces **F**^**m,z**^ (fig. S15C). The force **F**^**m,z**^ exerted on a particle is analyzed by considering the forces applied by its six neighboring particles (fig. S15B, inset), with a magnetic field strength of 10 mT, a field frequency of 10 Hz, and a representative field pitch angle of 60°. As shown in fig. S15D, in the oscillating magnetic field, the *z*-axial component of the magnetic field changes with time (the blue line), which results in the generation of time-varying force **F**^**m,z**^ (the red line). When the field strength is zero, the force **F**^**m,z**^ is zero (e.g., at 0.15 s). At this moment, the particle falls under gravity with a velocity of ~−4 μm/s (calculated using Eqs. 4 to 6), and it falls to a *z*-axial position of ~−15 nm (initially located at 0 nm), as shown in fig. S15E. Subsequently, the magnetic field strength increases, and, hence the force **F**^**m,z**^ increases to ~0.27 pN, pulling the particle upward with a velocity of ~6 μm/s. When the particle is approaching its initial position (i.e., getting closer to its neighboring particles), the force **F**^**m,z**^ decreases to ~0.11 pN, counterbalancing the gravitational force **F**^**g**^ (e.g., at 0.175 s). The velocity of the particle decreases to zero when the forces are in equilibrium, and the particle is retained in its initial position (~0 nm). Throughout the collective inclination process, the particle moves downward and upward regularly (fig. S15E), and its net displacement is approximately zero.

#### Part G: Asymmetrical oscillation of the collective

The oscillations of the collectives are asymmetric from experimental observations, due to the nature of the dual-axis oscillating fields. When two oscillating fields with different frequencies are applied, the resultant magnetic field exhibits asymmetries in the direction of the time-varying magnetic field vector, as shown in fig. S17 (A and B). At 0. 025 s, the field vector makes an angle of 81.4° with the *x* axis. Subsequently, it oscillates downward and makes an angle of −65.8° with the *x* axis at 0.075 s. In the next 0.05 s, the field vector oscillates upward and is deviated 54.7° from the *x* axis. Driven by the asymmetrical magnetic field, the oscillations of the collectives are periodically asymmetric, and the symmetry breaking leads to the drifting motions of the collectives (*37*, *45*). The average drifting speed of the collective is experimentally measured to be ~1 μm/s, and its drifting direction is unspecified given the same experimental condition. The unspecified drifting direction can be attributed to environmental factors such as the levelness of the container. As shown in fig. S17C, the collective drifts toward its left when the container is tilted by 0.5° (using a motorized stage), while it drifts toward its right when the container is tilted by −0.5°. The swirling motions of the particles inside the collectives and the streaming flows generated by the collectives are observed to be dependent on the direction of the asymmetrical collective oscillation. When the collective performs asymmetrical oscillation at its right side (fig. S17D), its composing particles swirl in the clockwise direction (Fig. 3B), and the streaming flow converges toward the left side of the collective and then rises toward the peak of the collective. In contrast, the streaming flow and particle swirling occur in the opposite direction when the collective oscillates asymmetrically at its left side. When the collective oscillates, clockwise and anticlockwise vortexes are generated one after another regularly, as simulated and shown in fig. S17E. The time-varying vortexes explain for the fluctuating motions of the tracers. As shown in fig. S17F, the tracers move upward and downward repeatedly, suggesting that they are subjected to the time-varying vortexes. Because of the asymmetrical collective oscillation, the velocity distribution of the vortexes is asymmetric, which leads to the generation of the streaming flow. Moreover, by tuning the pitch angle of the *x*-axial oscillating field, the direction of the asymmetrical collective oscillation can be adjusted, and directed motion of the collective can be achieved, as illustrated in fig. S18A. When a pitch angle is added, the profile of the magnetic field profile is asymmetric, and the collective performs asymmetrical up-down oscillation, as shown in fig. S18B and movie S5. When the pitch angle of the field is positive, the collective performs oscillation at its right side and moves to the right. In contrast, when the pitch angle of the field is negative, the collective oscillates at its left side and moves to the left. The influence of tuning the relative phases of the *x*-axial and *z*-axial oscillating fields on collective behaviors is investigated, as shown in fig. S24. In a high-frequency oscillating field (10 Hz), no notable change in collective behavior is observed when the phase shift is increased (fig. S24A). When phase shifts are added to the field, the oscillating angle and angular velocity of the magnetic field are affected. However, the collective is in a step-out state in the high-frequency field, and, hence, it cannot respond to the changes in the field. In contrast, when phase shifts are added to a low-frequency oscillating field (5 Hz), the collective performs translational motion, and its moving direction is dependent on the phase shifts of the field (fig. S24B). The motion of the collective is triggered because of the asymmetrical oscillation of the collective.

### Colloidal structures in different magnetic fields

Different magnetic fields are applied to induce self-assembly of the particles, and the results are summarized in table S1. The applied resultant field strength used in all groups is 20 mT; same particles (3 μm; ProMag HP, Bangs Laboratories) are used, and the areal particle concentrations are ~2 × 10^5^ particles/mm^2^ in all groups. When only the vertical interparticle interaction exists, particles form pillars with limited growth (fig. S7A). When only the vertical interparticle interaction and attractive interstructure interaction are induced, they form thicker and longer pillars, but their growth is still limited in the vertical direction (fig. S7B). When they are induced to have rotating behavior, they form rolling structures in which their rolling motions hinder interstructure assembly (fig. S7C). When our tailored magnetic field is applied, that is, the dual axis oscillating field, the particles form vertical collectives (fig. S7D).

### Controlled merging of the collectives

Merging of the vertical collectives can be controlled by reconfiguring the collective, as demonstrated in fig. S13. Initially, the field strength *A*, amplitude ratio γ, *x*-axial field frequency *f_x_*, and *z*-axial field frequency *f_z_* of the applied magnetic field are 10 mT, 1, 1 Hz, and 10 Hz, respectively. Four collectives (labeled with S1, S2, S3, and S4, respectively) are generated apart from each other. At 6 s, the axial direction of collective “S3” and that of collective “S4” (the green dashed lines) are tuned to coincide with the link between the centers of collective S3 and those of collective S4 (the red dashed line). Then, the amplitude ratio γ is decreased to 0.5, and collectives S3 and S4 are elongated (from 6 to 22 s). When the collectives are in contact with each other, they merge (at 22 s). By increasing the amplitude ratio γ to 1, the height of the merged structure becomes higher (from 22 to 35 s). To merge collective “S1,” collective “S2,” and the previously merged structure, the axial directions of the collectives are tuned to be parallel to the red link (at 35 s). Then, the amplitude ratio γ is again decreased to 0.5 to elongate the collectives for merging. From 79 to 88 s, the elongated structure is rotated to merge with collective S1 and collective S2. The amplitude ratio γ is then tuned to 1, and the merging of four small collectives into a single collective is realized (at 128 s).

### Simulation

Simulations on particle interactions, particle oscillations, collective generation, and collective hydrodynamic feature are conducted using the Fluid-Particle Interaction module in COMSOL Multiphysics package. Equation 1 is used to simulate the magnetic field. Equation 4 is used to simulate the magnetic dipole-dipole interaction force between particles. Equation 6 is used to simulate the gravitational force. Equation 8 is used to simulate the particle-particle excluded volume force. Equation 9 is used to simulate the repulsive force between the wall and the particles. The equations of drag force and volume force (exerted on fluids by the particles) in COMSOL are used. The parameters used in the simulation and analysis are summarized in table S2.
