# Supplementary material for: Gravity-resisting colloidal collectives
Source: Sci Adv. 2022 Nov 18;8(46):eade3161. doi: 10.1126/sciadv.ade3161 (PMC9674281; doi:10.1126/sciadv.ade3161)
Supplement: Supplementary file 1 — Figs. S1 to S24 Tables S1 and S2 [file sciadv.ade3161_sm.pdf]

Science Advances
AAAS

Supplementary Materials for

Gravity-resisting colloidal collectives

Junhui Law *et al.*

Corresponding author: Jiangfan Yu, yujiangfan@cuhk.edu.cn; Yu Sun, yu.sun@utoronto.ca

Sci. Adv. **8**, eade3161 (2022)
DOI: 10.1126/sciadv.ade3161

The PDF file includes:

Figs. S1 to S24
Tables S1 and S2
Legends for movies S1 to S5

Other Supplementary Material for this manuscript includes the following:

Movies S1 to S5

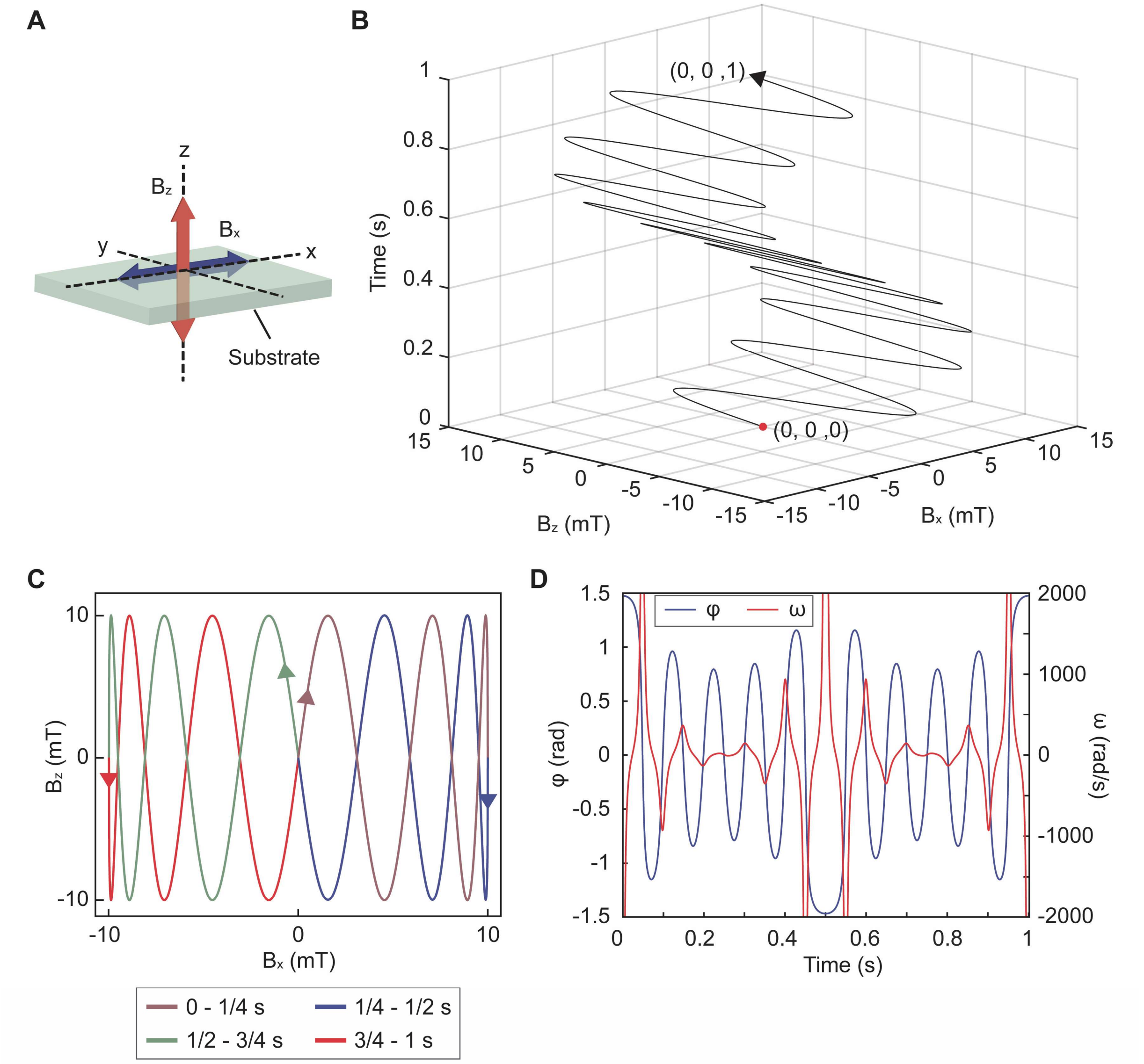

Fig. S1. Applied magnetic field. (**A**) The schematics showing the applied magnetic field. The blue and red arrows indicate the x-axial oscillating field B_x and z-axial oscillating field B_z, respectively. (**B**) The profile of the magnetic field with the field strength A, amplitude ratio γ, x-axial field frequency f_x, and z-axial field frequency f_z are 10 mT, 1, 1 Hz, and 10 Hz, respectively. The red dot indicates the point of the magnetic field at 0 s. (**C**) The detailed change in the magnetic field in a cycle. The brown, blue, green, and red curves indicate the changes of the magnetic field in the first, second, third, and fourth quarter of a cycle, respectively. (**D**) The change in the field oscillation angle $\varphi(t)$ and that of the field angular velocity $\boldsymbol{\omega}(t)$ with time in a cycle.

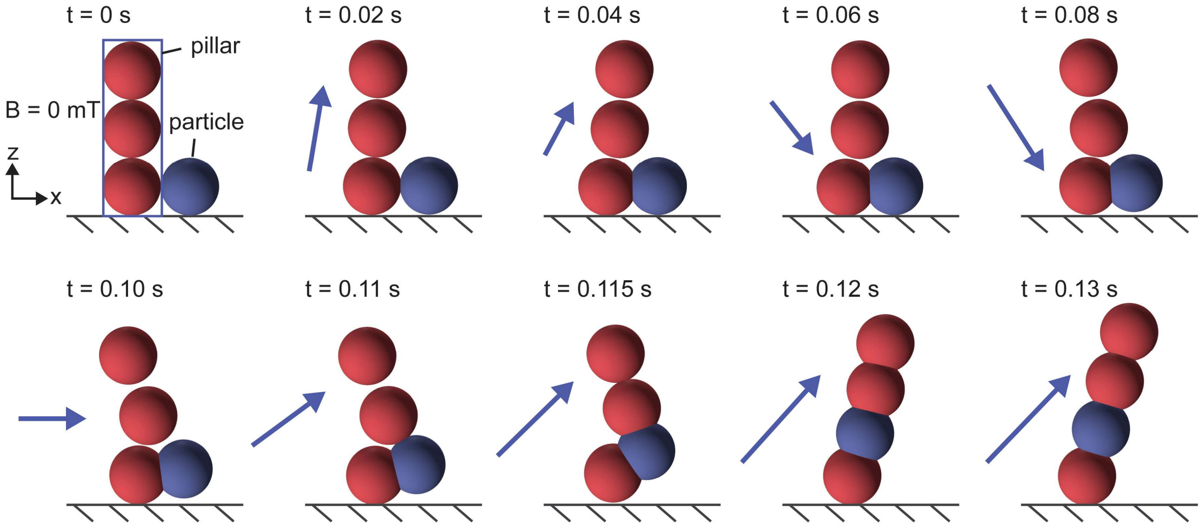

Fig. S2. Simulation results of colloidal pillar growth. The composing particles of the pillar are in red, and the single particle is in blue. The blue arrows represent the vectors of the magnetic field.

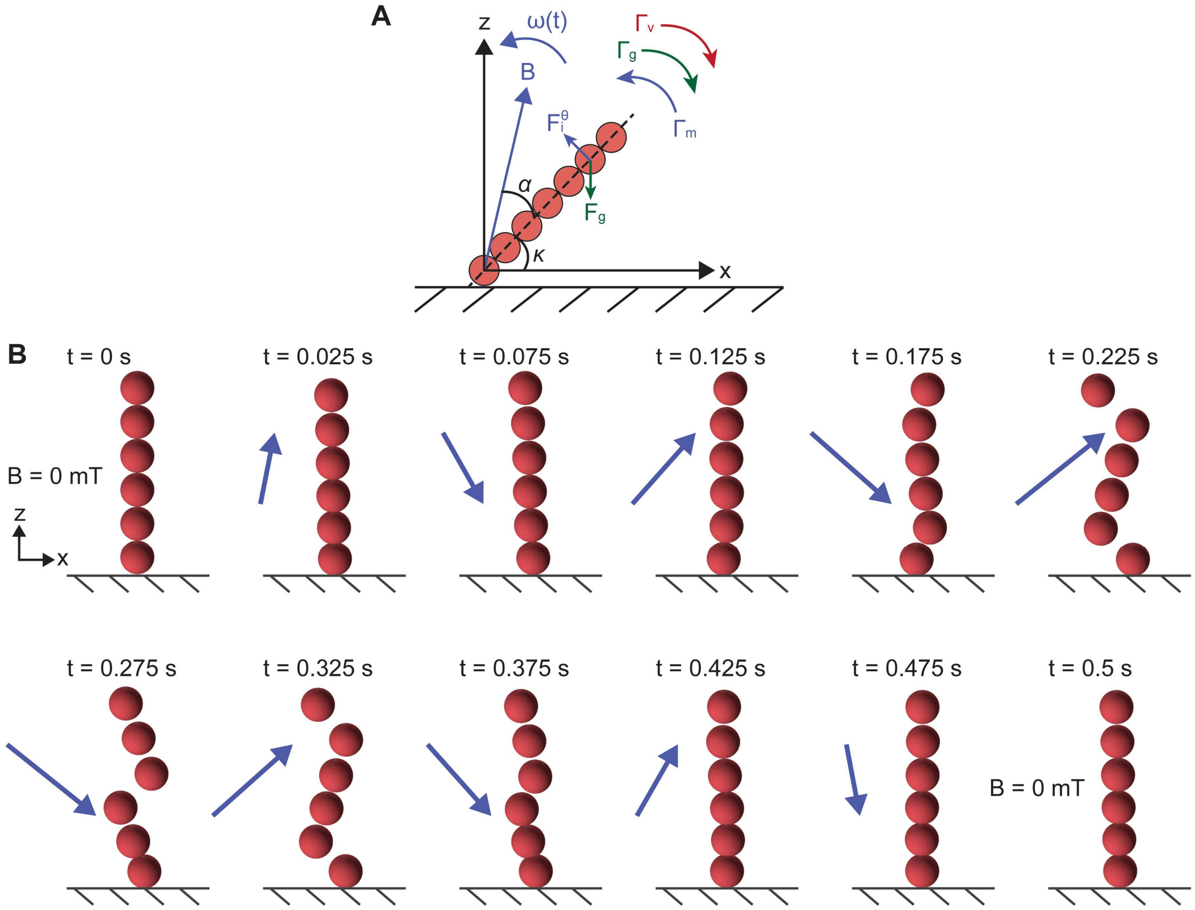

Fig. S3. Behavior of a colloidal pillar in the oscillating magnetic field. (**A**) The schematics analyzing the oscillation of a pillar in the oscillating magnetic field. The oscillating angle of a pillar is represented by κ. The angular velocity of the oscillating magnetic field is represented by $\boldsymbol{\omega}(t)$. The phase lag angle between the magnetic field and the long axis of the pillar is represented by α. The tangential component of the magnetic dipole-dipole interaction force exerted on particle i is represented by $\mathbf{F}_i^\theta$. The gravitational force is represented by $\mathbf{F}_g$. The magnetic torque, gravitational torque, and viscous torque exerted on the pillar are represented by $\boldsymbol{\Gamma}_m$, $\boldsymbol{\Gamma}_g$ and $\boldsymbol{\Gamma}_v$, respectively. (**B**) The simulated behavior of an oscillating pillar over time. The blue arrows represent the vectors of the magnetic field.

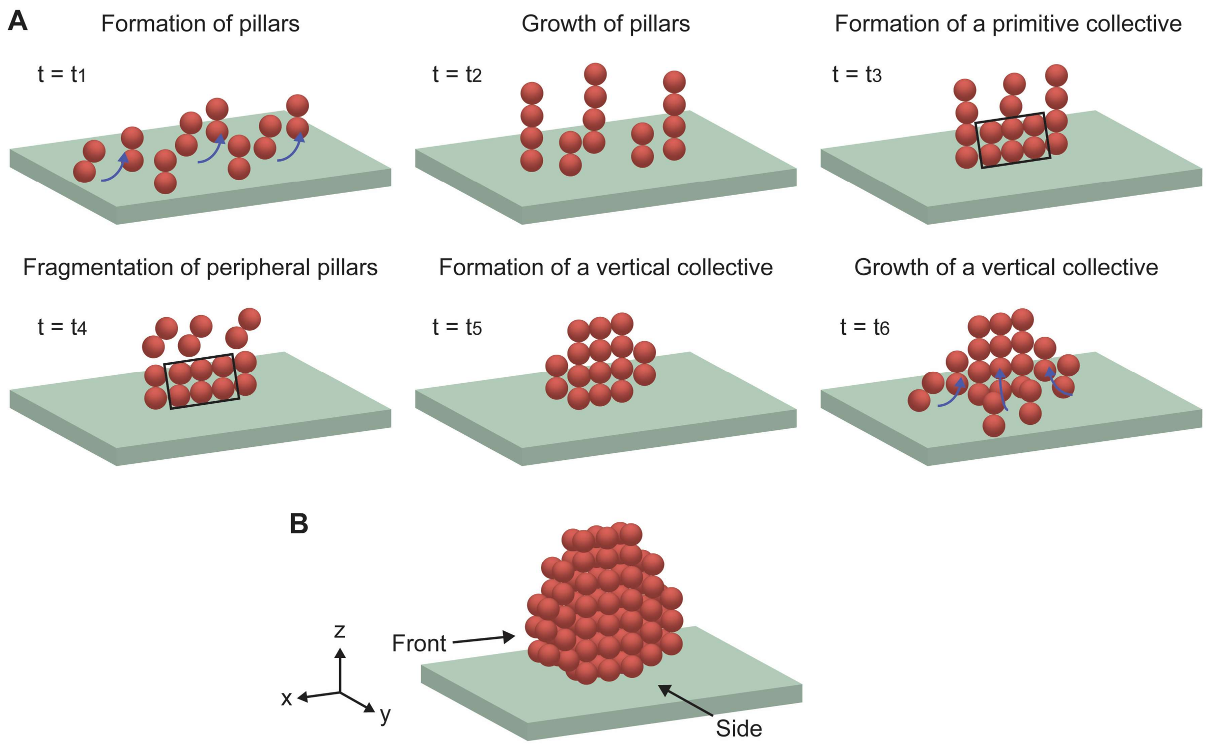

Fig. S4. Mechanism of the vertical collective generation. (**A**) The schematics showing the generation of a vertical collective. The blue arrows indicate the merging of the particles. The black boxes label the cores of the collective. (**B**) The schematics illustrating the front and side of the collective.

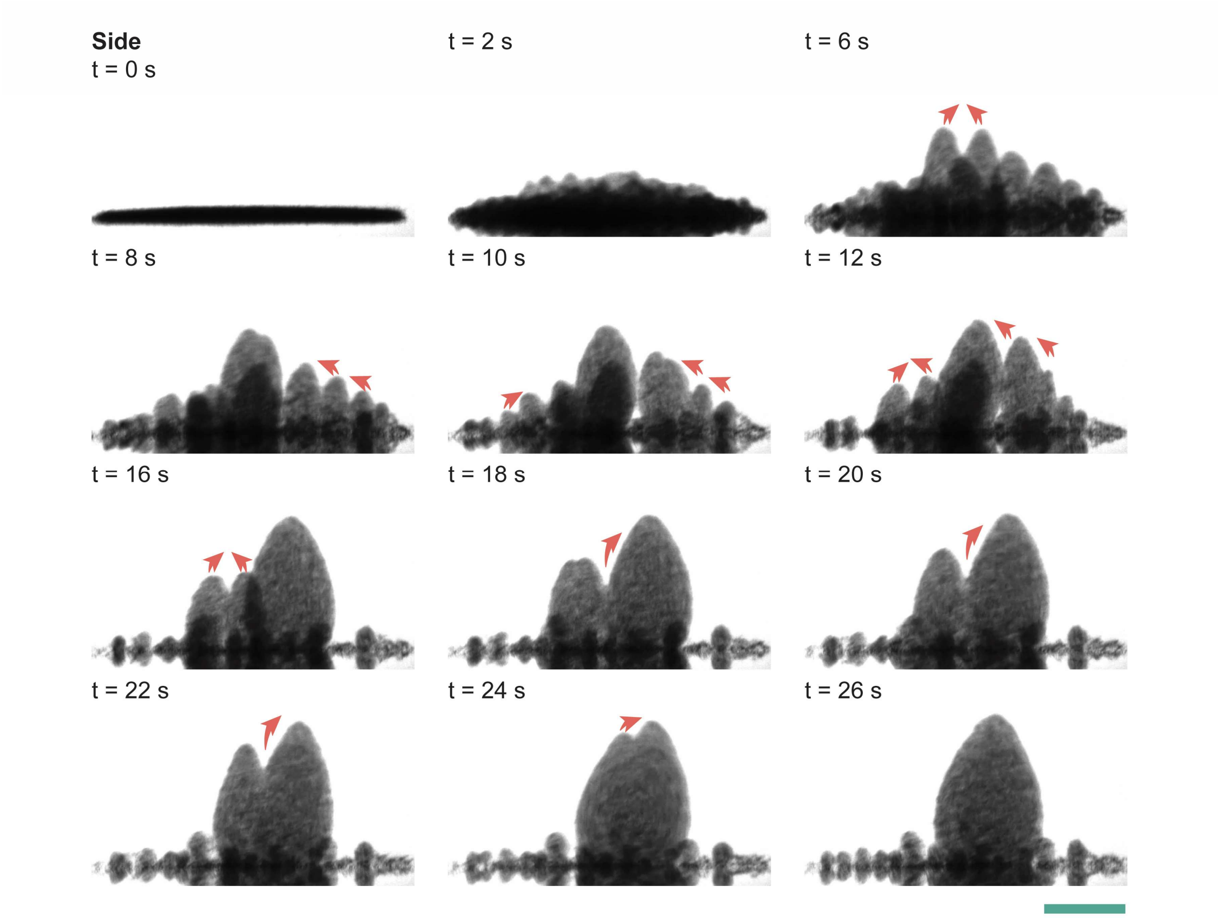

Fig. S5. Vertical collective generation. The red arrows indicate the merging behavior. The ‘side’ label indicates side view (projection onto x-z plane). Scale bar, 100 µm.

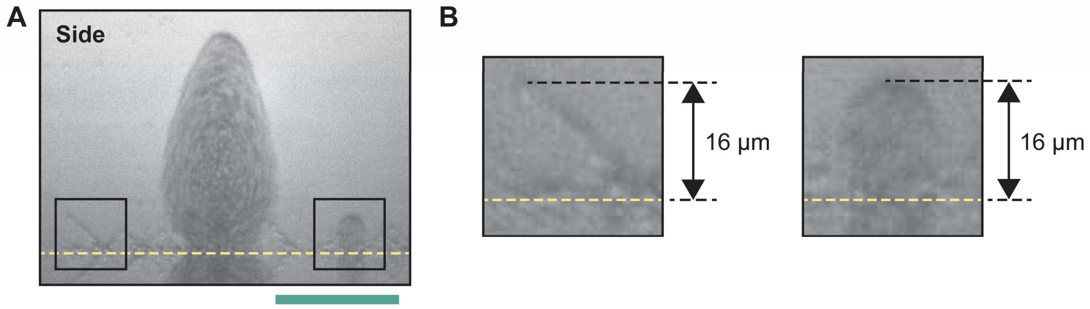

Fig. S6. Number of particles required for collective generation. (**A**) Observation of a colloidal pillar and collective consisting of approximately 8 and 75 units of particles, respectively. The applied magnetic field strength is 10 mT. The 'side' label indicates side view (projection onto x-z plane). Scale bar, 50 µm. (**B**) Zoom-in views of the pillar and the smallest collective in (A).

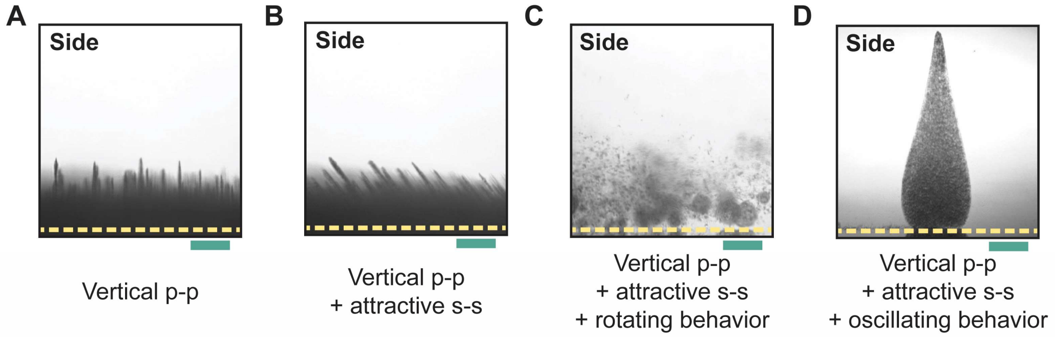

Fig. S7. Colloidal structures generated through different generation mechanisms. (**A**) Pillars. (**B**) Tilted pillars. (**C**) Rolling structures. (**D**) Vertical collective. The interactions between intermediate structures are indicated by using s-s while interparticle attractions are indicated by using p-p. The yellow dashed lines outline the substrate. The 'side' labels indicate side view (projection onto x-z plane). Scale bar, 100 μm.

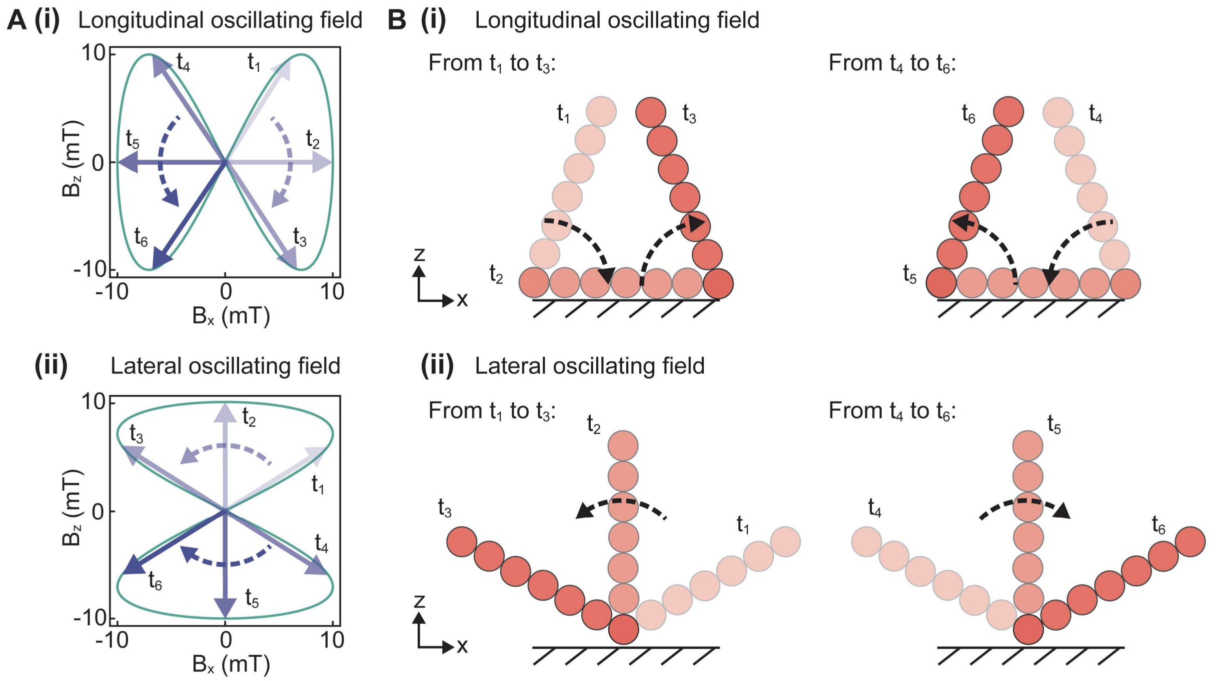

Fig. S8. Types of dual-axis oscillating magnetic fields. (**A**) Representative profiles of (i) longitudinal and (ii) lateral oscillating magnetic fields, respectively. B_z and B_x are the components of the magnetic field strength along the z-axis and x-axis, respectively. The blue arrows represent the vectors of the magnetic fields. The blue dashed arrows indicate the change in the direction of the field vectors with time t. The field strength A and amplitude ratio γ are 10 mT and 1. For the longitudinal oscillating field in (i), the x-axial field frequency f_x and z-axial field frequency f_z are 2 Hz and 4 Hz, respectively. For the lateral oscillating field in (ii), the x-axial field frequency and z-axial field frequency are 4 Hz and 2 Hz, respectively. (**B**) Schematics illustrating the oscillations of colloidal pillars in the representative (i) longitudinal and (ii) lateral oscillating fields, respectively. The black dashed arrows indicate the oscillating motions of the colloidal pillars.

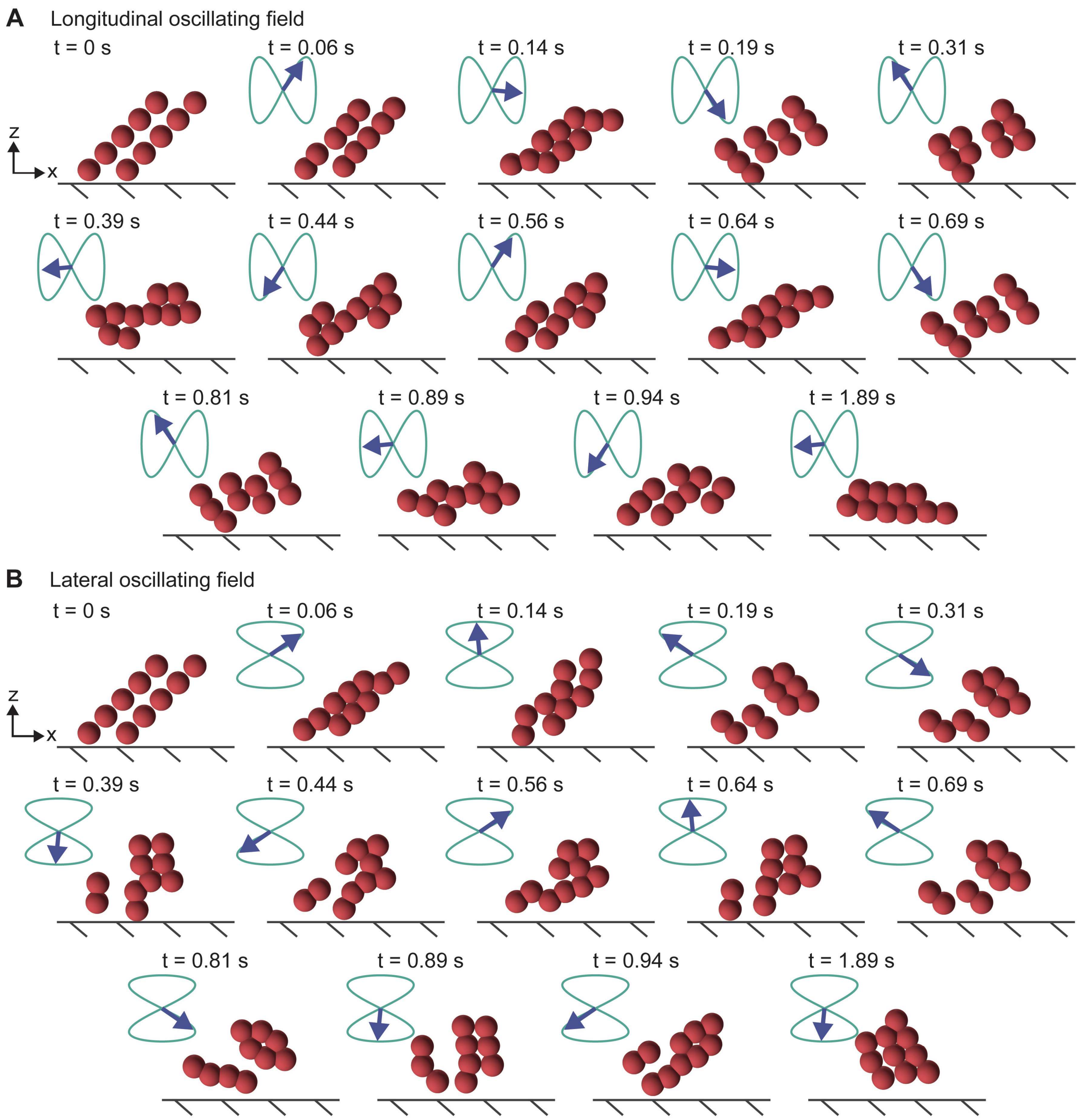

Fig. S9. Simulation results of colloidal pillar oscillation in different types of dual-axis oscillating magnetic fields. (**A**) Up-down oscillations of the pillars in the representative longitudinal oscillating field. (**B**) Side-to-side oscillations of the pillars in the representative lateral oscillating field. The blue arrows represent the vectors of the magnetic fields. The field strength A and amplitude ratio γ are 5 mT and 1, respectively. For the longitudinal oscillating field in (A), the x-axial field frequency f_x and z-axial field frequency f_z are 2 Hz and 4 Hz, respectively. For the lateral oscillating field in (B), the x-axial field frequency and z-axial field frequency are 4 Hz and 2 Hz, respectively.

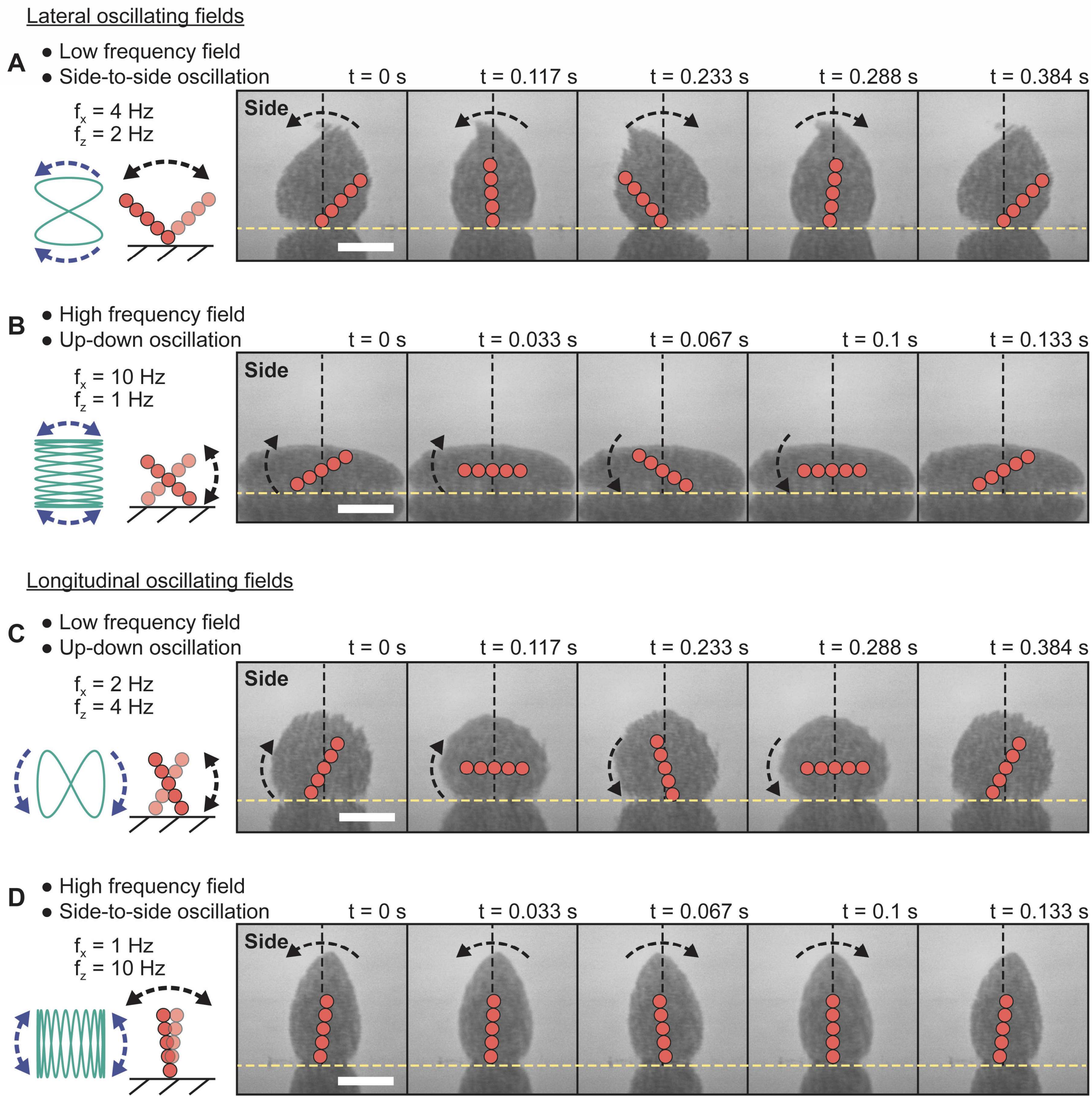

Fig. S10. Collective oscillation in different types of dual-axis oscillating magnetic fields. (**A** and **B**) Collective oscillation in the representative (A) low frequency and (B) high frequency lateral oscillating fields, respectively. (**C** and **D**) Collective oscillation in the representative low frequency (C) and (D) high frequency longitudinal oscillating fields, respectively. The inserted particle schematics in the experimental results represent the composing particles inside the collectives. The black dashed arrows indicate the oscillating motion of the composing particles in the collectives. The yellow dashed lines outline the substrates. The green curves represent the profiles of the magnetic fields, and the blue dashed arrows indicate the oscillating directions of the fields. The applied field strength A and amplitude ratio γ are 10 mT and 1. f_x and f_z are the x-axial and z-axial field frequencies of the applied oscillating fields, respectively. The 'side' labels indicate side view (projection onto x-z plane). Scale bars, 100 μm.

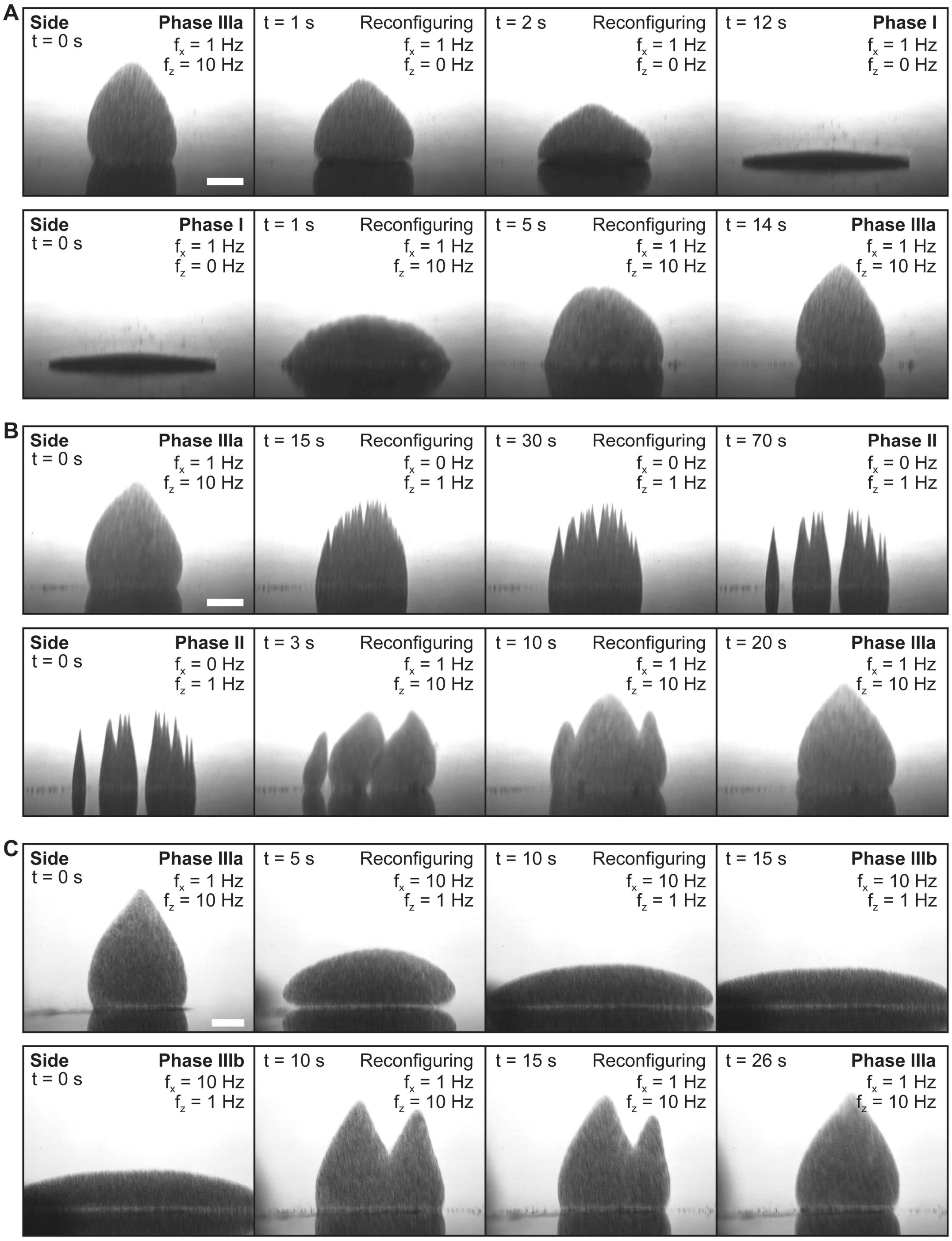

Fig. S11. Reversibility of the collectives in different phases. The collective reversibly changes between (**A**) Phase IIIa and Phase I; (**B**) Phase IIIa and Phase II; and (**C**) Phase IIIa and Phase IIIb. The applied field strength A and amplitude ratio γ are 10 mT and 1. f_x and f_z are the x-axial and z-axial field frequencies of the applied oscillating fields, respectively. The 'side' labels indicate side view (projection onto x-z plane). Scale bars, 100 µm.

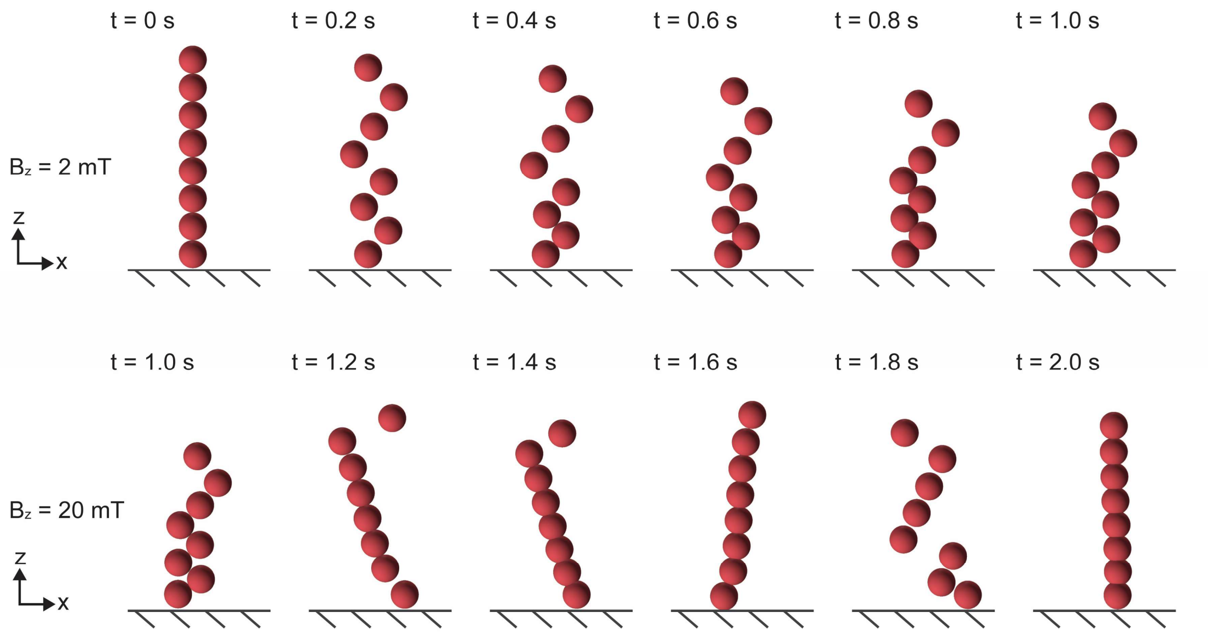

Fig. S12. Simulation results of the height reconfiguration of a colloidal pillar. The z-axial strength of the field is represented by B_z. The x-axial strength of the field is kept constant at 10 mT.

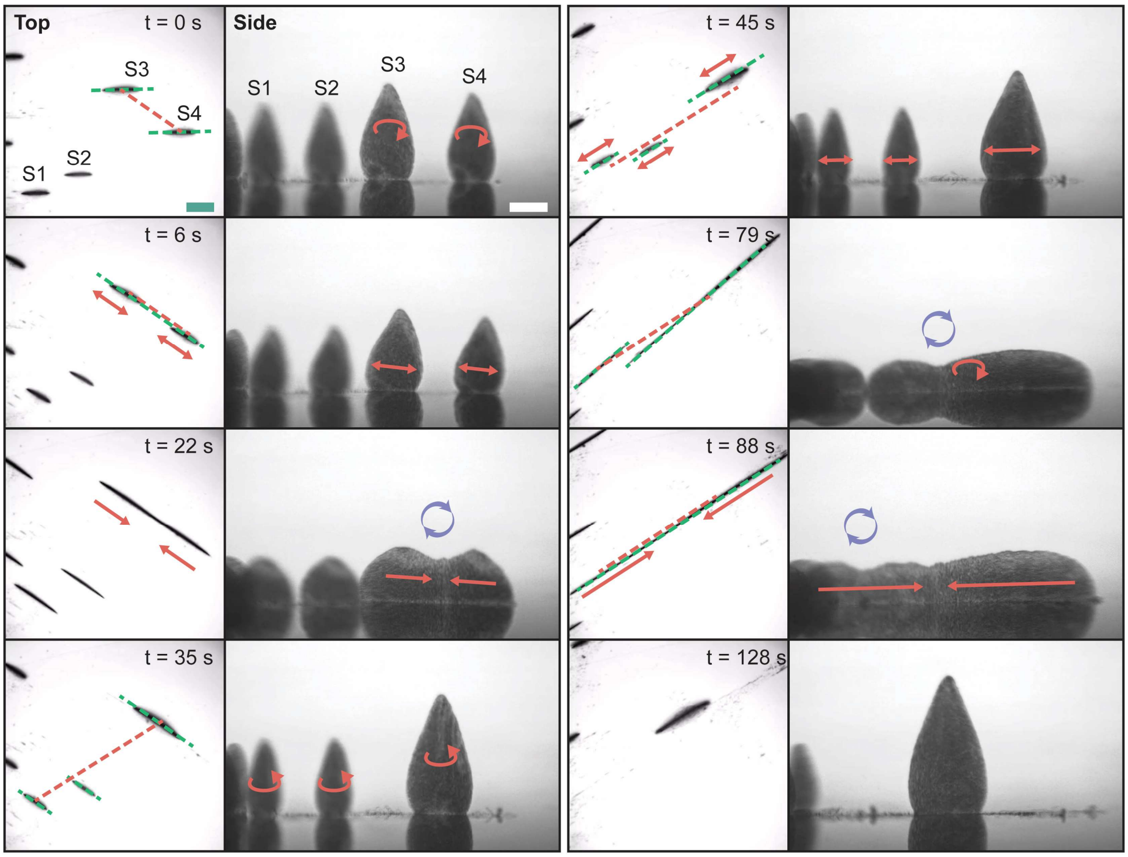

Fig. S13. Collective controlled merging. The frontal axes of the collectives are labelled by green dashed lines. The links between the center of two collectives are labelled by red dashed lines. The red arrows indicate the directions of elongation, contraction, and rotation of the collectives. The blue arrows indicate the merging of collectives. The ‘side’ and ‘top’ labels indicate side view (projection onto x-z plane) and top view (projection onto x-y plane), respectively. Scale bars, 100 μm.

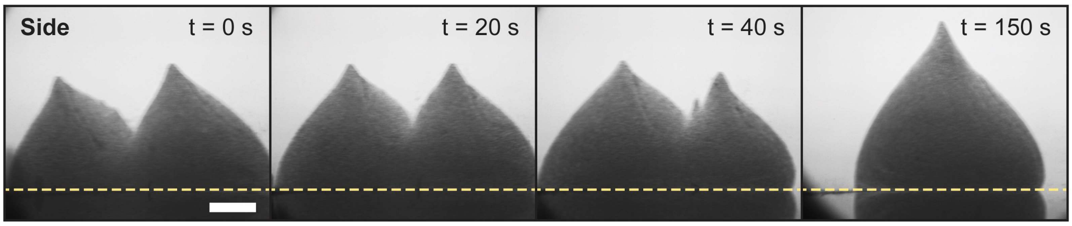

Fig. S14. Increased dimension of a collective through controlled merging. Generation of a collective with a height of 725 μm and length of 810 μm. The yellow dashed lines outline the substrates. The applied magnetic field strength is 10 mT, and the areal particle concentrations is ~ 2×10^5 particles per mm^2. The 'side' label indicates side view (projection onto x-z plane). Scale bar, 200 μm.

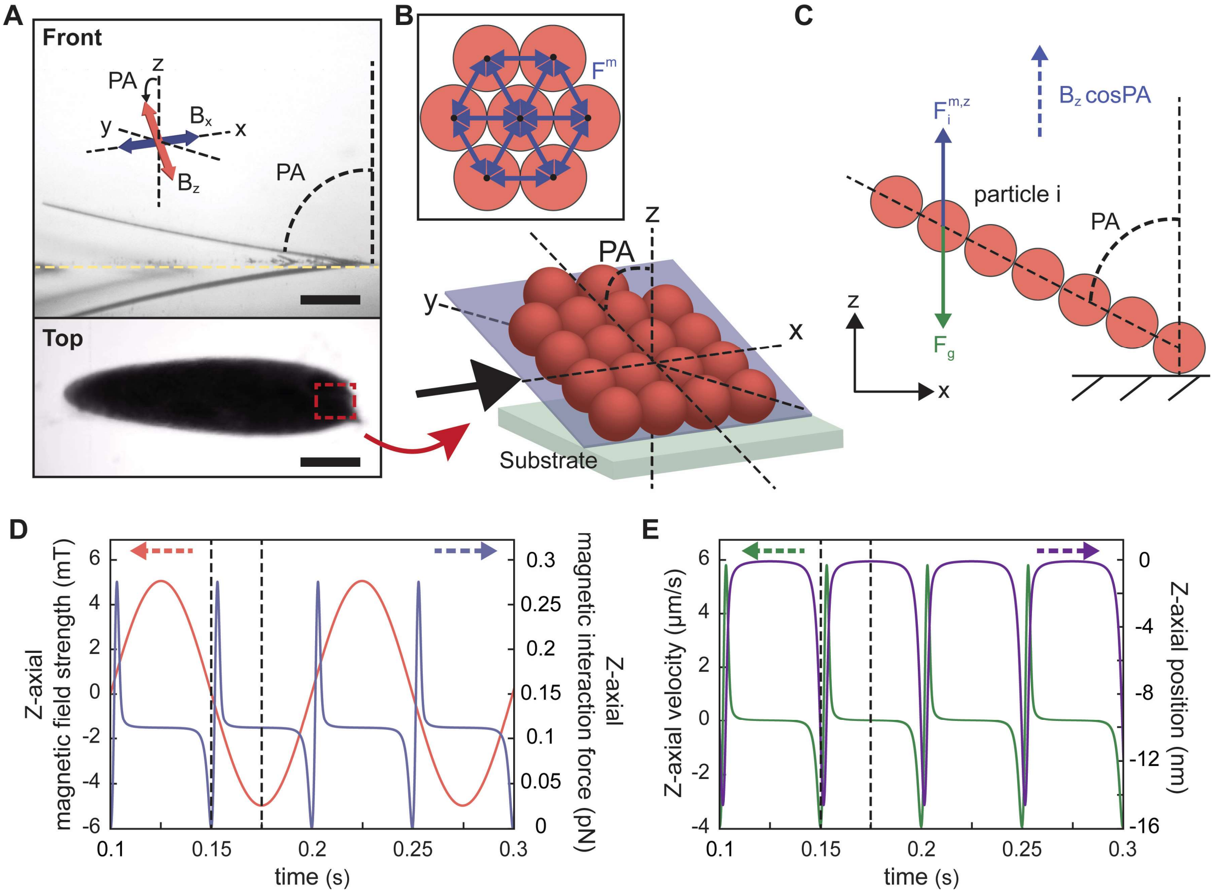

Fig. S15. Collective inclination. (**A**) Experimental results showing a tilted collective. The schematics show the applied magnetic field with the blue and red arrows indicating the x-axial oscillating field B_x and z-axial oscillating field B_z, respectively. PA is the pitch angle applied to the z-axial oscillating field B_z. The 'front' and 'top' labels indicate front view (projection onto y-z plane) and top view (projection onto x-y plane), respectively. Scale bars, 100 μm. (**B**) Schematics illustrating the composing particles of the tilted collective near the substrate. The blue region represents the oscillating plane of the particles. The inset shows the magnetic dipole-dipole interaction forces $\mathbf{F}^{m}$ between the particles. The blue arrows represent the force $\mathbf{F}^{m}$. (**C**) Schematics illustrating the forces acting on the particles. The view is projected from the front of the collective, as pointed by the black arrow in (B). $\mathbf{F}_{g}$ is the apparent weight of the composing particles. $\mathbf{F}^{m,z}$ is the z-axial components of the resultant interparticle magnetic interaction force. (**D**) The changes in the z-axial components of the magnetic field strength and those of the interparticle magnetic interaction force $\mathbf{F}^{m,z}$ with time. (**E**) The changes in the z-axial components of the particle velocity and those of the particle position with time.

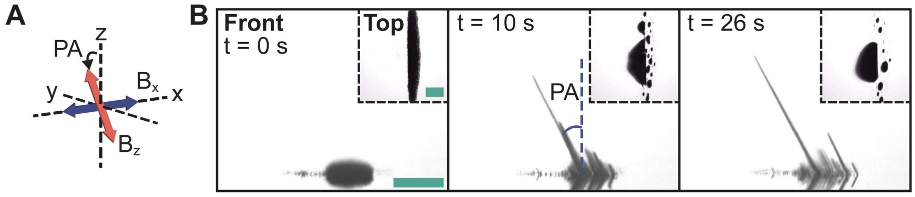

Fig. S16. Tilted generation of the collective. Dispersed particles directly self-assemble into tilted collectives upon energized by the magnetic field with a 30 º pitch angle. The schematics show the applied magnetic field with the blue and red arrows indicating the x-axial oscillating field B_x and z-axial oscillating field B_z, respectively. PA is the pitch angle applied to the z-axial oscillating field B_z. The 'front' and 'top' labels indicate front view (projection onto y-z plane) and top view (projection onto x-y plane), respectively. Scale bars, 100 µm.

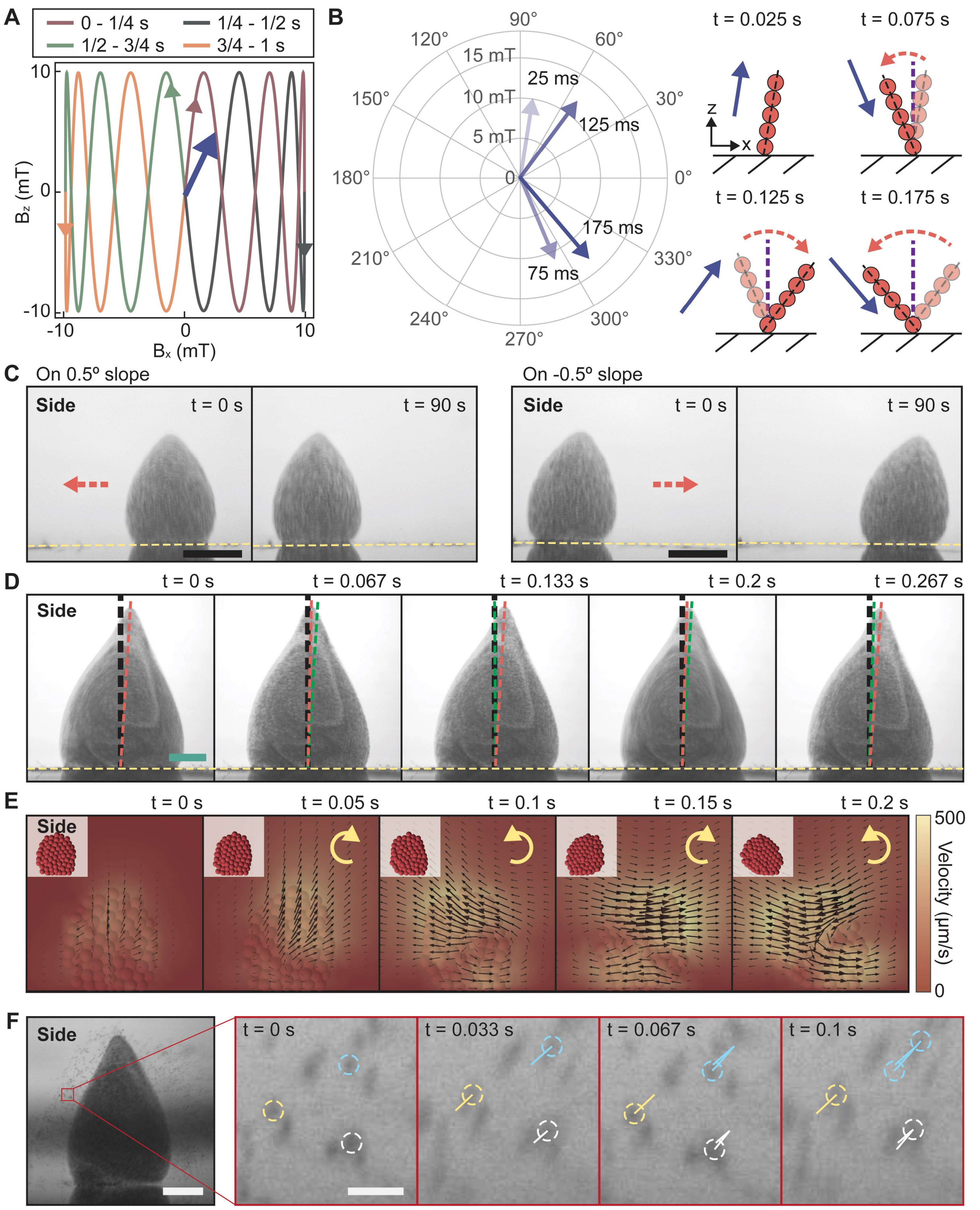

Fig. S17. Asymmetrical collective oscillation and flow generation. (**A**) Profile of the magnetic field showing the change in the z-axial and x-axial components of the magnetic field strength in a cycle. The blue arrow represents the vector of the magnetic field. The magnetic field strength *A*, amplitude ratio *γ*, x-axial field frequency f_x, and z-axial field frequency f_z are 10 mT, 1, 1 Hz, and

10 Hz, respectively. (**B**) Schematics showing the asymmetrical oscillations of particles. The polar plot shows the direction and magnitude of the vector of the magnetic field at different time points. The blue arrows represent the vector of the magnetic field. The purple dashed lines are vertical reference axes, and the red dashed arrows indicate the oscillating direction of the particles. (**C**) Drifting motion of the collective on slopes. The red dashed arrows indicate the drifting direction of the collective. Scale bars, 100 µm. (**D**) Experimental results showing the asymmetry in the collective oscillation. The black dashed lines are vertical reference axes. The red dashed lines are the axes intersecting with the peak of the collective and the center of the bottom part of the collective while the green dashed lines are the axes intersecting those of collective at previous time points. Scale bar, 100 µm. (**E**) The simulated hydrodynamic features of the collective. The black arrows represent the vectors of the flow field. The simulated collectives are shown on the top right corners. The yellow arrows indicate the directions of the vortexes. (**F**) Experimental results showing the fluctuating motions of the polystyrene tracing particles near the collective. Scale bars, 100 µm (left) and 10 µm (right). The 'side' labels indicate side view (projection onto x-z plane).

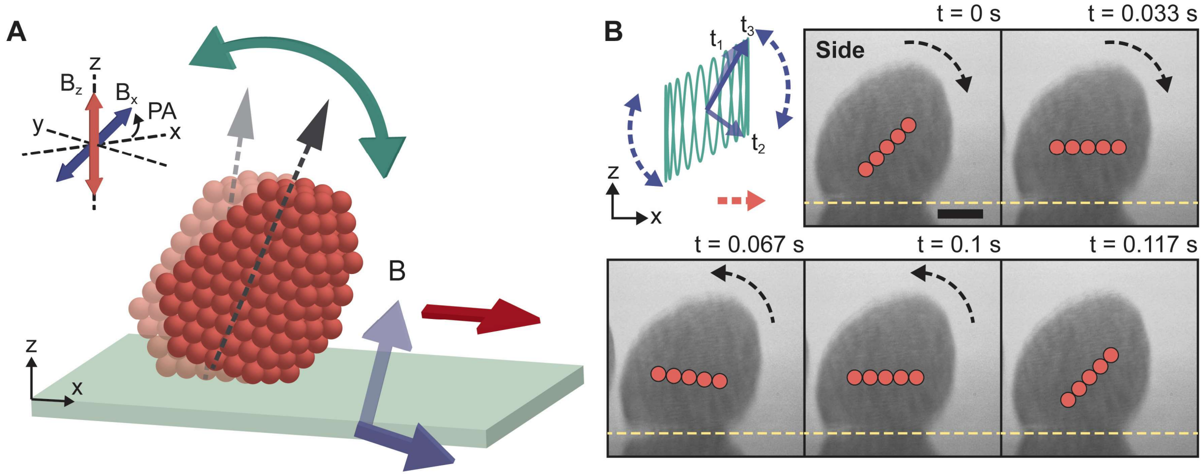

Fig. S18. Collective motion. (**A**) Schematics showing the directed asymmetrical oscillation of the collective. When a pitch angle PA is added to the x-axial oscillating field B_x, the vector of the magnetic field B oscillates asymmetrically. The red arrow indicates the moving direction of the collective, and the green arrow indicates the oscillating direction of the collective. (**B**) Experimental results showing the asymmetrical oscillation of the moving collective. The collective oscillation can be clearly observed in Movie S5. The schematics show the profile of the magnetic field with the pitch angle PA of 30º, field strength A of 10 mT, amplitude ratio γ of 1, x-axial field frequency f_x of 1 Hz, and z-axial field frequency f_z of 10 Hz. The black dashed arrows indicate the oscillating direction of the collective. The blue arrows represent the vectors of the magnetic field, and the blue dashed arrows indicate the oscillating direction of the magnetic field. The inserted particle schematics in the experimental results represent the composing particles inside the collectives. The red dashed arrow indicates the moving direction of the collective. Scale bar, 100 µm.

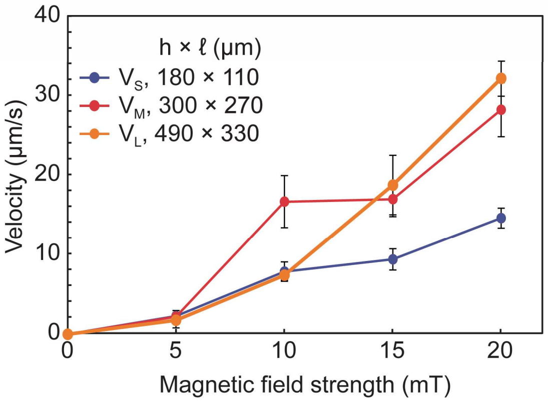

Fig. S19. Influence of magnetic field strengths on collective motion. The changes in the velocities of the collectives with the magnetic field strength. The first and second values in the legend indicate the heights and lengths of the small, medium, and large collectives, respectively when the applied magnetic field strength is 10 mT and the field pitch angle is 0 º. The velocities of the small, medium, and large collectives are represented by $\mathbf{V_S}$, $\mathbf{V_M}$, and $\mathbf{V_L}$, respectively. The field pitch angle is kept constant at 30 º. Each data point represents the average of three measurements. The error bars represent the standard deviations.

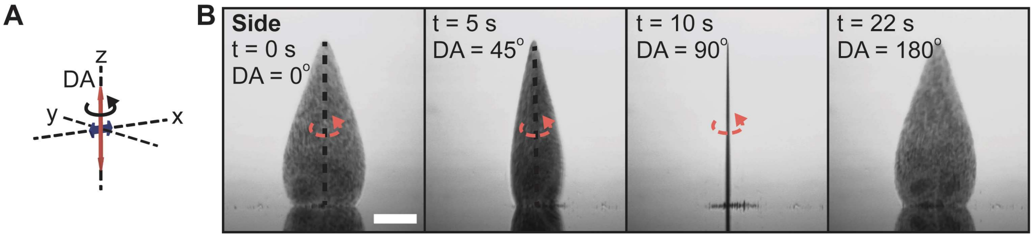

Fig. S20. Collective rotation. (**A**) The schematics showing the applied magnetic field. The blue and red arrows indicate the x-axial oscillating field B_x and z-axial oscillating field B_z, respectively. DA is the direction angle of the magnetic field. (**B**) The experimental results showing the rotation of a collective. The orientation of the collective is gradually rotated from 0 ° to 180 ° at an angular velocity of 0.16 rad/s. The red dashed arrows indicate the changes in the orientation of the collective. The 'side' label indicates side view (projection onto x-z plane). Scale bar, 100 μm.

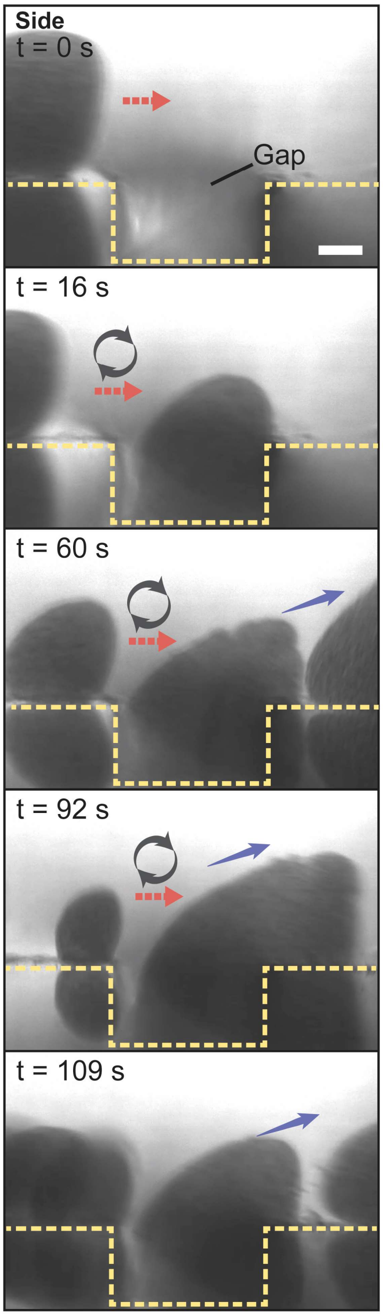

Fig. S21. Swarm behavior of the collectives. The collectives cooperate to pass through a gap with a width of 300 µm, which is wider than the length of the individual collective**.** The red arrows indicate the moving directions of the incoming collectives while the blue arrows indicate the moving directions of the departing collectives. The yellow dashed lines outline the substrate. The black arrows indicate the merging of the collectives. The 'side' label indicates side view (projection onto x-z plane). Scale bar, 100 µm.

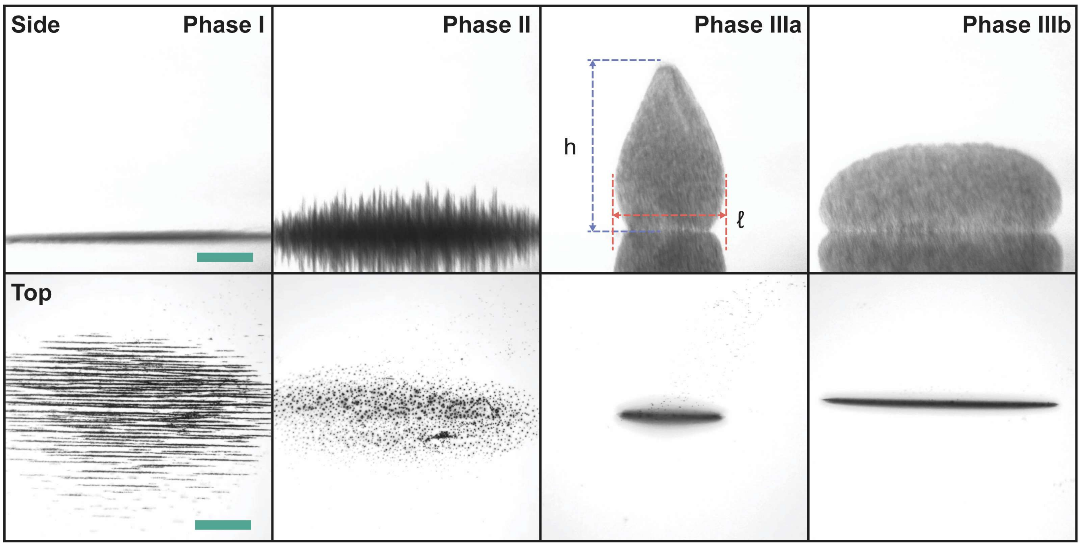

Fig. S22. Self-assembled colloidal structures. The side and top views of different phases of the colloidal structures. The height and length of the collectives are represented by *h* and *ℓ*. The 'side' and 'top' labels indicate side view (projection onto x-z plane) and top view (projection onto x-y plane), respectively. Scale bars, 100 μm.

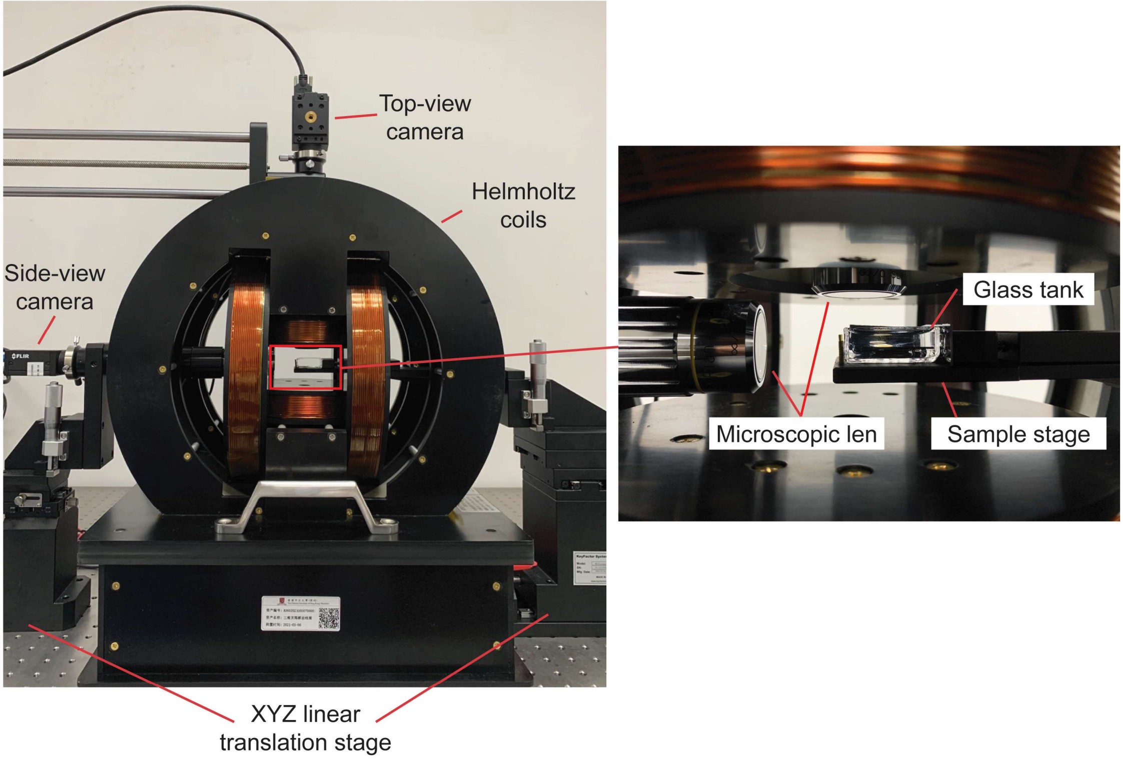

Fig. S23. Experimental setup. Three-axis Helmholtz electromagnetic coils are integrated with a top camera and a side camera. The sample glass tank is aligned with the top and side microscopic lens inside the coils.

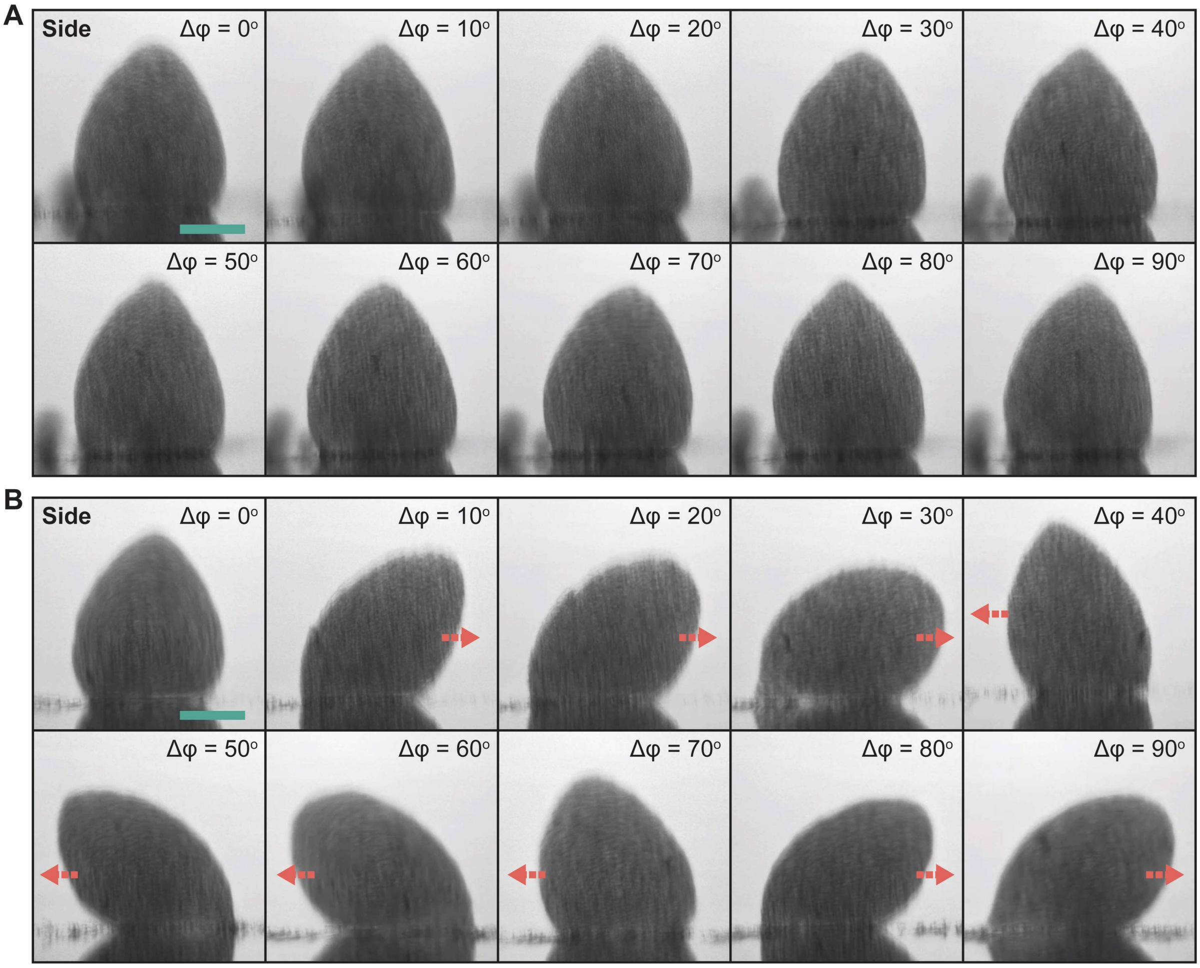

Fig. S24. Influence of field phase shift on collective behavior. (**A**) In a high frequency oscillating magnetic field with phase shifts Δφ. (**B**) In a low frequency oscillating magnetic field with phase shifts Δφ. The field strength A and amplitude ratio γ are 10 mT and 1. For the applied field in (A), the x-axial field frequency f_x and z-axial field frequency f_z are 1 Hz and 10 Hz, respectively. For the applied field in (B), the x-axial field frequency f_x and z-axial field frequency f_z are 1 Hz and 5 Hz, respectively. Δφ represents the phase shift of the magnetic fields. The red dashed arrows indicate the motions of the collectives. The 'side' labels indicate side view (projection onto x-z plane). Scale bars, 100 μm.

Table S1. Colloidal structures generated using different magnetic fields.

Types of magnetic fields*		Vertical interparticle attraction	Attractions among intermediate structures	Reconfig-urability[#]	Vertical growth	Observation (side view)
Uniform field	In xy plane	-	-	-	-	-
	Out of xy plane	Yes	-	-	Limited	Pillar
Single axis oscillating field	In xy plane	-	-	-	-	-
	Out of xy plane	Yes	-	-	Limited	Pillar
Conical field	Rotation axis in xy plane	Yes	Yes	-	Limited	Pillar
	Rotation axis out of xy plane	Yes	Yes	-	Limited	Pillar
Rotating field	In xy plane	-	Yes	Yes	-	-
	Out of xy plane	Yes	Yes	Yes	Limited	Rolling structure
**Dual-axis oscillating field**	In xy plane	-	Yes	Yes	-	-
	**Out of xy plane**	**Yes**	**Yes**	**Yes**	**Yes**	**Vertical collective**

[#]Reconfigurability indicates the positional reconfigurability of the assembled particles.
*The equations of magnetic fields are listed below.

- The equation of the uniform field is $\mathbf{B} = A\hat{\mathbf{e}}$, where A is the magnetic field strength, and $\hat{\mathbf{e}}$ is the unit vector.
- The equation of the single axis oscillating field is $\mathbf{B}(t) = A\sin(2\pi ft)\hat{\mathbf{e}}$, where f is frequency, and t is time.
- The equation of the rotating field is $\mathbf{B}(t) = A\sin(2\pi ft)\hat{\mathbf{e}}_\mathrm{n} + A\cos(2\pi ft)\hat{\mathbf{e}}_\mathrm{m}$, where $\hat{\mathbf{e}}_\mathrm{n}$ and $\hat{\mathbf{e}}_\mathrm{m}$ are unit vectors, and the angle between the unit vectors is 90 degrees.
- The equation of the conical field is $\mathbf{B}(t) = A\cos(W)\sin(2\pi ft)\hat{\mathbf{e}}_\mathrm{n} + A\cos(W)\cos(2\pi ft)\hat{\mathbf{e}}_\mathrm{m} + A\sin(W)\hat{\mathbf{e}}_\mathrm{n} \times \hat{\mathbf{e}}_\mathrm{m}$, where W is the semi-cone angle.

Table S2. Parameters used in simulation and analysis.

Particle diameter	D	3×10^{-6} [m]
Particle magnetic susceptibility	χ_r	0.3
Particle density	ρ_r	1800 [kg/m^3]
Free space permeability	μ_0	1.257×10^{-6} [kgm/s^{2}A^{-2}]
Fluid viscosity	η	1.0×10^{-3} [Pas]
Fluid density	ρ_f	1000 [kg/m^3]
X-axial magnetic field strength	B_x	2×10^{-3} to 20×10^{-3} [T]
Z-axial magnetic field strength	B_z	2×10^{-3} to 20×10^{-3} [T]
X-axial magnetic field frequency	f_x	1 to 10 [Hz]
Z-axial magnetic field frequency	f_z	1 to 10 [Hz]
Interparticle collision diameter	σ_r	3×10^{-6} [m]
Interparticle interaction strength	ε_r	2×10^{-20} [J]
Wall-particle collision diameter	σ_w	1.5×10^{-6} [m]
Wall-particle interaction strength	ε_w	1×10^{-30} [J]

Captions for Movies

Movie S1. Generation and gravity-resisting characteristics of the vertical collectives
This video sequentially shows the self-assembly, reconfiguration, collapse, inclination, tilted self-assembly, and controlled merging of the collectives (corresponding to Fig. 1A, 2A, 2D to 2F, and fig. S13, respectively).

Movie S2. Self-assembly mechanism of the vertical collectives
This video sequentially shows the self-growing and oscillating behavior of a colloidal pillar, and the self-assembly of a vertical collective.

Movie S3. Adaptive locomotion of the vertical collectives
This video shows the collectives are navigated to perform locomotion along a circular path, on a slope and in non-zero fluidic flow environment (corresponding to Fig. 4A to 4C, respectively). It further shows a collective is reconfigured to adapt into and pass through a confined channel (corresponding to Fig. 4D).

Movie S4. Swarm behavior of the vertical collectives
This video shows the collectives cross an obstacle and a gap, and they collectively climb a stair and cross a wide gap (corresponding to Fig. 4E to 4G, and fig. S21, respectively). It further shows the collectives work together to cover the light path in a circular microchannel (corresponding to Fig. 4H).

Movie S5. Supplementary information on collective oscillation
This video shows the oscillations of the collectives in different types of oscillating fields (corresponding to fig. S10). It further shows the oscillation of a moving collective (corresponding to fig. S18B).
